# Transcriptome analysis reveals major transcriptional changes during regrowth after mowing of red clover *(Trifolium pratense*)

**DOI:** 10.1186/s12870-021-02867-0

**Published:** 2021-02-15

**Authors:** Denise Brigitte Herbert, Thomas Gross, Oliver Rupp, Annette Becker

**Affiliations:** 1 Justus Liebig University, Institute of Botany, Heinrich-Buff-Ring 38, D-35392 Giessen, Germany; 2 Department of Bioinformatics and Systems Biology, Justus Liebig University, Heinrich-Buff-Ring 26-32, D-35392 Giessen, Germany

**Keywords:** *Trifolium pretense*, Red clover, RNAseq, Regrowth reaction, Biotic and abiotic stress, Field conditions, Gibberellins

## Abstract

**Background:**

Red clover (*Trifolium pratense*) is globally used as a fodder plant due its high nutritional value and soil improving qualities. In response to mowing, red clover exhibits specific morphological traits to compensate the loss of biomass. The morphological reaction is well described, but the underlying molecular mechanisms and its role for plants grown in the field are unclear.

**Results:**

Here, we characterize the global transcriptional response to mowing of red clover by comparing plants grown under greenhouse conditions with plants growing on agriculturally used fields. Unexpectedly, we found that biotic and abiotic stress related changes of plants grown in the field overlay their regrowth related transcriptional changes and characterized transcription related protein families involved in these processes. Further, we can show that gibberellins, among other phytohormones, also contribute to the developmental processes related to regrowth after biomass-loss.

**Conclusions:**

Our findings show that massive biomass loss triggers less transcriptional changes in field grown plants than their struggle with biotic and abiotic stresses and that gibberellins also play a role in the developmental program related to regrowth after mowing in red clover. Our results provide first insights into the physiological and developmental processes of mowing on red clover and may serve as a base for red clover yield improvement.

**Supplementary Information:**

The online version contains supplementary material available at 10.1186/s12870-021-02867-0.

## Background

*Trifolium pratense* (red clover) is an important worldwide forage crop and thus of great economic interest. This perennial plant offers several advantages like a high protein content and soil improving characteristics, which reduce the use of artificial nitrogen fertilizer and enhance livestock intake. Well-known disadvantages of red clover include poor persistence under several land use scenarios, like grazing or cutting [1–3]. *T. pratense* is a member of the Fabaceae (or legumes), which are, due to their economic value, among the most examined families in the plant kingdom with genome sequences available for species like *Medicago truncatula* (barrel clover) [4], *Lotus japonicus* (birdsfoot trefoil) [5], *Glycine max* (soy) [6], *Phaseolus vulgaris* (common bean) [7], *Cicer arietinum* (chickpea) [8], *Vigna unguiculata* (cowpea) [9], *Trifolium subterraneum* (subterranean clover) [10] *T. medium* (zigzag clover) [11], and *T. pratense* (red clover) [12, 13].

Facing today’s challenges such as an increased demand on food production in an era of global climate change, together with the aim to solve these problems in an environmental friendly and sustainable way, requires improvement of forage crops like *T. pratense* [14, 15]. *T. pratense* breeding aims to offer genotypes with improved key agronomic traits (dry matter yield, high quality, resistance to diseases and abiotic/biotic stress, persistency), while improving its regrowth ability [2, 16]. Unfortunately, the morphological investigations of several *T. pratense* populations showed a correlation of persistency with non-favorable traits, like small plant size and prostrate growth habit [17]. Moreover, most *T. pratense* cultivars or accessions are locally adapted and require their specific local conditions to show the favored traits [18, 19], which decreases the stability for individual traits in breeding efforts [20]. *T. pratense* exhibits significant intraspecific variation due to high intrapopulation genetic diversity, thus, persistence and performance in response to mowing or cutting depends on the variety as well as on the developmental stage at the moment of damage [21–24].

Persistency can be defined as a sustained forage yield over several growing periods [25] and is a complex trait influenced by a variety of abiotic and biotic factors, and the regrowth ability of a plant [26]. Plants with high regrowth ability can survive more frequent and intense biomass loss and are therefore more persistent. Decapitation or biomass loss due to herbivory or mowing triggers a complex reaction affected by environmental conditions, plant morphology, architecture, developmental stage and genotype [21]. After decapitation, the first known stress response in other legumes like *M. sativa* and *P. sativum* involves the production of phytohormones: cytokinins, auxins, and strigolactones [27–29]. In addition, the mobilization of energy reserves is activated [30]. Phenotypic plasticity of plant architecture in combination with alterations of hormone concentrations can be observed in *P. sativum* and *T. pratense* after decapitation [24, 29, 31]. However, the molecular processes allowing plants to thrive even after an enormous loss of biomass remain still unclear, even in *Arabidopsis thaliana* [32, 33].

Here, we compare the transcriptomes of mown (cut) vs. unmown (uncut) *T. pratense* plants from two different, well investigated field locations on the Biodiversity Exploratory “Heinich-Dün” [34] and greenhouse grown plants. Our field samples were subjected to standard agricultural treatment and we can thus discriminate transcriptional changes caused by abiotic factors and biotic interactions in the field from those that regulate regrowth. We present the identification and in silico characterization of putative developmental regulators differentially expressed in the regrowth phase after mowing in the field and in the greenhouse that may contribute to the regrowth response of *T. pratense* and demonstrate that gibberellin is a major regulator of specific aspects of the regrowth morphology in red clover.

## Results

### RNA-Seq results, de novo assembly, and functional description of contigs

The RNA-Seq produced a total number of short reads between 44.7 and 58.1 million for each library with two exceptions (Table S4) totaling 608,041,012 raw reads. The de novo assembly of the reference transcriptome of *T. pratense* produced 44,643 contigs, of which 41,505 contigs were annotated and 29,781 contigs were identified as plant specific. The minimal length of the contigs was 124 bp, the maximal length 15,551 bp (Table S5). After the de novo assembly of the *T. pratense* transcriptome, each library was mapped back against the reference transcriptome to determine the overall alignment rate, which was between 77.85 and 90.32% (Table S6).

63% of the 44,643 contigs could be mapped to a known locus of the *T. pratense* genome annotation [12, 35], 32% could be mapped to an unknown locus of the *T. pratense* genome and 5% could not be mapped to the *T. pratense* genome (Fig. S2). All plant-specific contigs were annotated with several databases (Table S3). To further verify the quality of our replicates, we identified the transcripts shared by the two replicates. We calculated TPM values for each transcript and discarded transcripts with TPM values < 1. The percentage of transcripts shared between the two replicates was between 90 and 94% for all treatments/localities, suggesting that the RNA-Seq data are highly reproducible (Table S8). In addition, we validated the RNA-Seq data via q-RT-PCR of four randomly chosen contigs tdn_146439 (LTP), k65_9861 (P5CS), tdn_69411 (PME44), and tdn_85889 (ENGase85A). The expression pattern of all four genes was congruent in qRT-PCR and RNA-Seq samples (Fig. S3). When single tissues (axillary meristem (AM) and leaves (L) were tested individually, only LTP showed expression similar to the RNA-Seq data.

### Differentially expressed gene analysis reveals diverse subsets of genes involved in regrowth influenced by location and environmental conditions

To identify gene expression responses underlying the regrowth response after mowing, a digital gene expression analysis was performed comparing field A mown vs. field A non-mown (FaM vs. FaNM); field B mown vs. field B non-mown (FbM vs. FbNM); greenhouse mown vs. greenhouse non-mown (GM vs. GNM) to identify DEGs (Table S9) from mown plants. Interestingly, using the |log2foldchange| ≥ 2, the number of differentially expressed genes (DEGs) is rather similar in all comparisons, ranging from 119 (GM vs. GNM) to 142 (FaM vs. FaNM) (Table 1).

**Table 1:** The numbers of differentially expressed transcripts (contigs) between libraries with changes equal or above |log2foldchange| 2. Upregulation for each comparison is shown

Analysis	Total DEGs	Number of upregulated transcripts in mown libraries	Number of upregulated transcripts in unmown libraries
GM vs. GNM	119	54	65
FaM vs. FaNM	142	49	93
FbM vs. FbNM	122	59	63

### GO enrichment

We performed a GO enrichment analysis with the DEGs of each group to obtain a differential view on the transcriptional changes occurring in relation to regrowth (Tables 2, 3, 4). The identified GO terms for each sample (FaM, FbM, GM, FaNM, FbNM, and GNM) were then compared in a mown vs. non-mown manner to identify GO terms specific for the respective treatments. The results revealed that GO terms involved mainly in general metabolic processes and pathways, as well as general reactions are enriched in non-mown plants including i.e. the GO terms “protein metabolic process”, “metabolic process”, “cellular process”, “catabolic process”, “biosynthetic process” (Fig. S4 and Table S11). Within mown samples we found the following GO terms enriched: “nucleic acid binding “(GM); GO terms related to photosynthesis (“photosynthesis”, “thylakoid“), cell components and protein transport (“Golgi apparatus”, “cytoplasm“) and related to regrowth and stress response (“generation of precursor metabolites and energy”, “cell growth”, “cell communication“) (Fig. S4 and Table S11). Within the GO term “cell growth” the contigs GIBBERELLIN-REGULATED PROTEIN 1 which is involved in cell elongation and ROOT HAIR DEFECTIVE 3, a protein involved in root hair growth are present. For FbM we found the GO term related to metabolic processes (“metabolic process”, “lipid metabolic process”), cell related (“cytoplasm”, “extracellular space”), enzymatic and catabolic processes (“enzyme regulator activity”, “catalytic activity”) and the GO term “binding”, which included a contig encoding for “V”, a protein involved in the ethylene biosynthesis.

**Table 2:** Twenty most strongly differentially expressed genes of the GM vs. GNM analysis. Shown are the transcript name, |log2foldchange| ≥ 2 of the corresponding transcript, the library in which the transcript is upregulated (pattern), gene name based on *T. pratense* genome annotation, corresponding Phytozome description, gene name and species name of the next homologs and *A. thaliana* gene name, and locus name based on information available on Tair

ID	Pattern	Contig ID	|log2foldchange| ≥ 2	Class (basis of classifiation)	Gene name ***T. pratense***	Next homolog gene name	Next homolog species name	***A. thaliana*** gene name	***A. thaliana*** locus name
**1**	GHNM	tdn_99733	−9.5	Growth (*M. truncatula*)	Tp57577_TGAC_v2_mRNA4544.v2	Medtr4g029550.1	*M. truncatula*	*–*	–
**2**	GHNM	k41_54584	−6.3	Biotic stress (*T.pratense, M. truncatula, A. thaliana*)	Tp57577_TGAC_v2_mRNA28349.v2	Medtr5g073620.1	*M. truncatula*	*ATEXO70B1*	AT5G58430
**3**	GHNM	tdn_92791	−5.5	Abiotic/biotic stress (*T.pratense, M. truncatula, A. thaliana*)	Tp57577_TGAC_v2_mRNA20498.v2	Medtr1g041150.1	*M. truncatula*	*ATCPK1*	AT5G04870
**4**	GHNM	k41_130218	−5.5	–	–	–	*–*	*–*	–
**5**	GHNM	tdn_53091	−4.8	Phytohormone (*M. truncatula, A. thaliana*)	Tp57577_TGAC_v2_mRNA39912.v2	Medtr4g010250.1	*M. truncatula*	*–*	AT5G20190
**6**	GHNM	tgg_43136	−4.4	Transcription (*M. truncatula, A. thaliana*)	Tp57577_q_v2_mRNA29629.v2	Medtr4g098630.1	*M. truncatula*	*ANAC071*	AT4G17980
**7**	GHNM	tdn_141837	−4.3	Abiotic stress (*T.pratense, M. truncatula, A. thaliana*)	Tp57577_TGAC_v2_mRNA760.v2	Medtr2g022700.1	*M. truncatula*	*ATGPT2*	AT1G61800
**8**	GHNM	tdn_40997	−4.2	Abiotic stress (*T.pratense, M. truncatula, A. thaliana*)	Tp57577_TGAC_v2_mRNA25718.v2	Medtr4g130540.1	*M. truncatula*	*HSP70B*	AT1G16030
**9**	GHNM	k71_5292	−4.1	Biotic stress (*T.pratense, M. truncatula*)	Tp57577_TGAC_v2_mRNA23166.v2	Medtr0163s0020.1	*M. truncatula*	*LECRK-IX.1*	AT5G10530
**10**	GHNM	k59_6358	−3.9	Growth (*T.pratense, M. truncatula, A. thaliana*)	Tp57577_TGAC_v2_mRNA12337.v2	Medtr3g435430.1	*M. truncatula*	*ATEXP15*	AT2G03090
**11**	GHM	tdn_86219	8.0	Biotic stress (*T.pratense, M. truncatula, A. thaliana*)	Tp57577_TGAC_v2_mRNA29036.v2	Medtr4g066210.1	*M. truncatula*	*BGLU12*	AT5G42260
**12**	GHM	k23_115785	8.0	Abiotic stress (*T.pratense, M. truncatula, A. thaliana*)	Tp57577_TGAC_v2_mRNA22071.v2	Glyma.01G001000.1	*G. max*	*–*	AT5G58110
**13**	GHM	tdn_91159	8.1	Biotic stress (*T.pratense, M. truncatula, A. thaliana*)	Tp57577_TGAC_v2_mRNA7745.v2	Medtr4g035870.1	*M. truncatula*	*–*	AT5G62360
**14**	GHM	k65_43517	8.3	Phytohormone (*T.pratense, A. thaliana*)	Tp57577_TGAC_v2_mRNA6281.v2	Medtr1g082750.1	*M. truncatula*	*ATAMI1*	AT1G08980
**15**	GHM	tgg_18067	8.4	–	Tp57577_TGAC_v2_mRNA32019.v2	–	–	*–*	–
**16**	GHM	k61_38813	9.0	–	–	–	–	*–*	–
**17**	GHM	k49_82496	9.0	Abiotic/biotic stress (*G. max, A. thaliana*)	Tp57577_TGAC_v2_mRNA37976.v2	Glyma.06G268800.1	*G. max*	*–*	AT4G04790
**18**	GHM	k67_38815	9.1	Biotic stress (*T.pratense*)	Tp57577_TGAC_v2_mRNA41666.v2	Medtr0062s0020.1	*M. truncatula*	*–*	–
**19**	GHM	k45_11164	9.6	Transcription (*T.pratense*)	Tp57577_TGAC_v2_mRNA29953.v2	Medtr3g092510.1	*M. truncatula*	*ATRBP37*	AT4G10610
**20**	GHM	tdn_25484	9.6	Growth (*Phaseolus vulgaris*)	Tp57577_TGAC_v2_mRNA13093.v2	Phvul.006G033800.1	*Phaseolus vulgaris*	*–*	–

**Table 3:** Twenty most strongly differentially expressed genes of the FaM vs. FaNM analysis. Shown are the transcript name, |log2foldchange| ≥ 2 of the corresponding transcript, the library in which the transcript is upregulated (pattern), gene name based on *T. pratense* genome annotation, corresponding Phytozome description, gene name and species name of the next homologs and *A. thaliana* gene name, and locus name based on information available on Tair

ID	Pattern	Contig ID	|log2foldchange| ≥ 2	Class (basis of classifiation)	Gene name *T. pratense*	Next homolog gen name	Next homolog species name	*A. thaliana* gene name	*A. thaliana* locus name
1	TPNM2	k33_17052	−9,0	Biotic stress (*T. pratense, M. truncatula, A. thaliana*)	Tp57577_TGAC_v2_mRNA21474.v2	Medtr4g079440.1	*M. truncatula*	na	AT1G06260
2	TPNM2	k43_111792	−8,8	Biotic stress (*M. truncatula*)	Tp57577_TGAC_v2_mRNA26333.v2	Medtr8g101900.1	*M. truncatula*	CCOAOMT7	AT4G26220
3	TPNM2	tdn_34568	−8,6	–	Tp57577_TGAC_v2_mRNA9104.v2	Glyma.13G061800.1	*G. max*	–	AT5G39530
4	TPNM2	tdn_49640	−8,6	–	–	–	*–*	–	–
5	TPNM2	tdn_58745	−8,5	Biotic stress (*T. pratense, M. truncatula, A. thaliana*)	Tp57577_TGAC_v2_mRNA20190.v2	Medtr8g075200.1	*M. truncatula*	–	AT1G75900
6	TPNM2	tdn_47209	−8,5	Growth (*M. truncatula, A. thaliana*)	Tp57577_TGAC_v2_mRNA10703.v2	Medtr1g053315.1	*M. truncatula*	–	AT1G03390
7	TPNM2	tdn_48478	−8,4	Biotic stress (*T. pratense, M. truncatula, A. thaliana*)	Tp57577_TGAC_v2_mRNA19516.v2	Medtr2g099020.1	*M. truncatula*	–	AT3G59510
8	TPNM2	k41_17597	−8,4	Growth stress (*T. pratense, M. truncatula, A. thaliana*)	Tp57577_TGAC_v2_mRNA8526.v2	Medtr1g036490.1	*M. truncatula*	ATCOMT, ATOMT1	AT5G54160
9	TPNM2	k51_82581	−8,2	Growth (*T. pratense*)	Tp57577_TGAC_v2_mRNA23127.v2	Medtr2g436480.1	*M. truncatula*	KCS21	AT5G49070
10	TPNM2	tdn_82424	−8,1	Growth (*T. pratense*)	Tp57577_TGAC_v2_mRNA17103.v2	Medtr2g013740.1	*M. truncatula*	KCS10	AT2G26250
11	TPM2	k49_380	7,5	Development (*A. thaliana*)	Tp57577_TGAC_v2_mRNA37185.v2	SapurV1A.0885 s0040.1	*Salix purpurea*	DAYSLEEPER	AT3G42170
12	TPM2	tdn_49869	7,6	–	–	–	*–*	–	–
13	TPM2	tdn_54983	7,7	–	–	–	*–*	–	–
14	TPM2	k37_9029	7,8	–	–	–	*–*	–	–
15	TPM2	k45_6120	8,4	–	Tp57577_TGAC_v2_mRNA2166.v2	Medtr2g007510.1	*M. truncatula*	–	–
16	TPM2	k71_23808	8,4	Development (*T. pratense, M. truncatula, A. thaliana*)	Tp57577_TGAC_v2_mRNA14131.v2	Medtr1g021320.1	*M. truncatula*	–	AT4G33280
17	TPM2	k59_3541	8,4	Development (*T. pratense, M. truncatula, A. thaliana*)	Tp57577_TGAC_v2_mRNA34193.v2	Medtr4g089030.1	*M. truncatula*	CYP71A26	AT3G48270
18	TPM2	k59_360	8,6	Metabolism (*Linum usitatissimum, T. pratense*)	Tp57577_TGAC_v2_mRNA21875.v2	Lus10012445	*Linum usitatissimum*	–	AT1G50020
19	TPM2	k53_38903	9,0	Abiotic stress (*A. thaliana*)	Tp57577_TGAC_v2_mRNA37328.v2	Medtr8g063190.1	*M. truncatula*	PRIN2	AT1G10522
20	TPM2	tdn_129978	9,6	–	Tp57577_TGAC_v2_mRNA9318.v2	Medtr7g062280.1	*M. truncatula*	–	AT5G01140

**Table 4:** Twenty most strongly differentially expressed genes of the FbM vs. FbNM analysis. Shown are the transcript name, |log2foldchange| ≥ 2 of the corresponding transcript, the library in which the transcript is upregulated (pattern), gene name based on *T. pratense* genome annotation, corresponding Phytozome description, gene name and species name of the next homologs and *A. thaliana* gene name, and locus name based on information available on Tair

ID	Pattern	Contig ID	|Log2foldchange| ≥ 2	Class (basis of classifiation)	Gene name ***T. pratense***	Next homolog gen name	next homolog species name	***A. thaliana*** gene name	***A. thaliana*** locus name
**1**	TPNM3	tdn_100726	−9,4	Biotic stress (*T. pratense, M. truncatula, A. thaliana*)	Tp57577_TGAC_v2_mRNA24659.v2	Medtr4g094772.1	*M. truncatula*	*CYP81D*	AT4G37340
**2**	TPNM3	tgg_49631	−8,0	Biotic stress (*T. pratense, M. truncatula, A. thaliana*)	Tp57577_TGAC_v2_mRNA37846.v2	Medtr6g034470.1	*M. truncatula*	*–*	AT2G34930
**3**	TPNM3	tdn_152262	−7,9	–	–	–	–	*–*	–
**4**	TPNM3	tdn_56712	−7,9	Biotic stress (*T. pratense, M. truncatula*)	Tp57577_TGAC_v2_mRNA30556.v2	Medtr8g027540.1	*M. truncatula*	*–*	–
**5**	TPNM3	tdn_87762	−7,9	Biotic stress (*T. pratense, M. truncatula, A. thaliana*)	Tp57577_TGAC_v2_mRNA10533.v2	Medtr7g451400.1	*M. truncatula*	*ATMCP1B, ATMCPB1*	AT1G02170
**6**	TPNM3	tdn_86129	−7,1	General cell functions (*T. pratense, M. truncatula, A. thaliana*)	Tp57577_TGAC_v2_mRNA10207.v2	Glyma.11G154500.1	*G. max*	*RPB5E*	AT3G54490
**7**	TPNM3	k55_46241	−6,9	Growth (*T.pratense, M. truncatula, A. thaliana*)	Tp57577_TGAC_v2_mRNA31452.v2	Medtr4g128150.1	*M. truncatula*	*histone 4*	AT2G28740
**8**	TPNM3	tdn_55533	−6,2	Abiotic stress (*T. pratense, M. truncatula, A. thaliana*)	Tp57577_TGAC_v2_mRNA39263.v2	Medtr5g007790.1	*M. truncatula*	*ATCRM1, ATXPO1*	AT5G17020
**9**	TPNM3	tgg_51443	−4,7	Growth (*T. pratense, M. truncatula, A. thaliana*)	Tp57577_TGAC_v2_mRNA37076.v2	Medtr5g019580.2	*M. truncatula*	*UGT72E2*	AT5G66690
**10**	TPNM3	tdn_136706	−4,7	–	–	–	–	*–*	–
**11**	TPM1	tdn_140636	8,8	General cell functions (*T. pratense, M. truncatula, A. thaliana*)	Tp57577_TGAC_v2_mRNA28209.v2	Medtr8g005980.1	*M. truncatula*	*C-NAD-MDH2*	AT5G43330
**12**	TPM1	tdn_154158	8,9	General cell functions (*T. pratense, M. truncatula*)	Tp57577_TGAC_v2_mRNA39482.v2	Medtr3g114970.2	*M. truncatula*	*–*	AT5G55150
**13**	TPM1	tdn_65187	9,1	Transposon (*T. pratense, Prunus persica A. thaliana*)	Tp57577_TGAC_v2_mRNA30115.v2	Prupe.4G011200.1	*Prunus persica*	*–*	AT4G29090
**14**	TPM1	tdn_100956	9,2	Metabolism (*T. pratense, Capsella rubella, A. thaliana*)	Tp57577_TGAC_v2_mRNA9542.v2	Carubv10008027m	*Capsella rubella*	*AHA2*	AT4G30190
**15**	TPM1	k63_21505	9,3	Biotic stress (*T. pratense, M. truncatula, A. thaliana*)	Tp57577_TGAC_v2_mRNA19467.v2	Medtr3g022400.1	*M. truncatula*	*–*	AT3G14470
**16**	TPM1	tdn_142681	9,3	Secondary metabolite biosynthesis (*T. pratense, M. truncatula, A. thaliana*)	Tp57577_TGAC_v2_mRNA15473.v2	Medtr8g074550.1	*M. truncatula*	*–*	AT2G18570
**17**	TPM1	k45_6120	9,6	–	Tp57577_TGAC_v2_mRNA2166.v2	Medtr2g007510.1	*M. truncatula*	*–*	–
**18**	TPM1	tdn_52922	10,1	–	Tp57577_TGAC_v2_mRNA41271.v2	mrna20290.1-v1.0-hybrid	*Fragaria vesca*	*–*	AT1G21280
**19**	TPM1	tdn_65185	10,9	–	–	–	–	*–*	–
**20**	TPM1	tdn_109277	11,7	Transcription (*T. pratense, M. truncatula, A. thaliana*)	Tp57577_TGAC_v2_mRNA29560.v2	Medtr5g028610.1	*M. truncatula*	*–*	AT3G14460

**Fig. 1:**
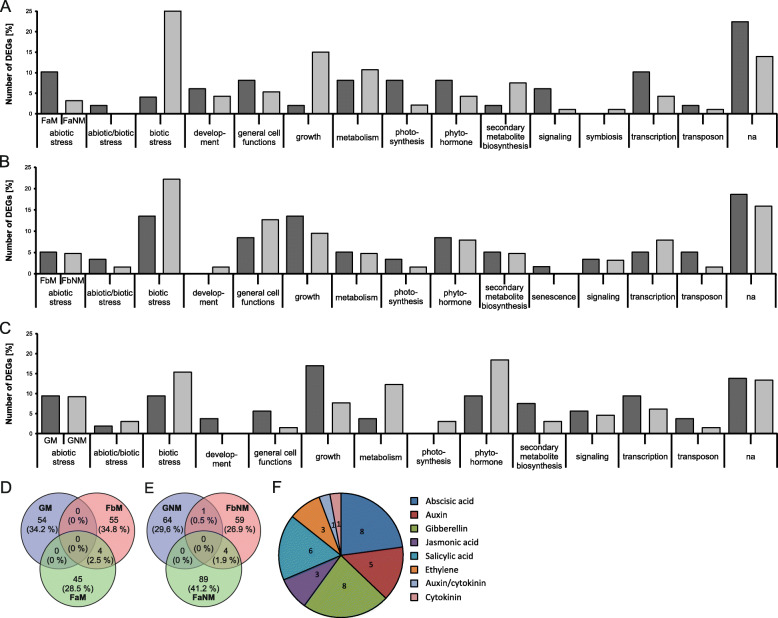
Transcriptome analysis showing biological processes relevant after mowing. **a**-**c** DEGs with a |log2foldchange| ≥ 2 are shown in percentage share of each class to the corresponding DEG list in bar charts, dark grey bars show datasets from mown, light grey bars indicate datasets from non-mown plants, showing the changing distribution of DEGs in the various biological processes after mowing. **a** Classes of DEGs from field A, **b** Classes of DEGs from field B, **c** Classes of DEGs from greenhouse grown plants. **d**-**e** Venn diagrams showing the number of shared upregulated genes within the mown samples (**d**) and the number of shared genes within the unmown samples (**e**) with a |log2foldchange| ≥ 2. Blue circles indicate genes upregulated in the greenhouse, green indicates genes upregulated in field A, and red indicates genes upregulated in field B. **f** Number of DEGs belonging to the class “phytohormones” within the DEG list of the two field and the greenhouse transcriptomes. The pie chart shows the number of DEGs related to the different phytohormones (abscisic acid, auxin, genes common between the auxin and cytokinin pathway, cytokinin, ethylene, gibberellins, jasmonic acid, and salicylic acid)

Interestingly, most functional groups differ between the field and greenhouse location (Fig. 1a-c), for example, more genes related to growth are upregulated in the non-mown Fa location but in the Fb and greenhouse location, they more genes are upregulated in the mown plants. Only genes related to biotic stress processes were upregulated in all unmown locations and more transposon-related genes are upregulated in mown plants (Fig. 1 a-c).

The photosynthesis- and phytohormone-related genes of field A show a similar pattern to the field B plants as do the phytohormone- and signaling related genes. Genes related to development, general cell functions and transcription are also similar between field A and the greenhouse grown plants, such that more transcription- and development-related genes are upregulated in mown plants. And unexpectedly, senescence-related genes are upregulated in mown plants of field B. However, as our analysis cannot discriminate between activating and repressing factors of senescence, we cannot conclude from our data whether the mown plants have activated or repressed their senescence program.

The largest group of differentially expressed genes is the one related to biotic stress with up to 38% differentially expressed genes in one location (field B, Fig. 1 b). This suggests that biotic stresses play a prominent role in non-mown plants. A similar phenomenon can be observed for growth related processes, where up to 24% genes were upregulated in the mown and unmown plants indicating that different growth programs are active in mown vs. unmown plants.

Taken together we can state that mown plants in all locations change their transcriptional programs upon mowing suggesting that they massively change their metabolism and signaling processes. However, the molecular answer to substantial biomass loss differed between all three locations.

To find similarly regulated genes between the treatments and/or locations, Venn diagrams were generated to compare the number of shared DEGs within the mown samples and the non- mown samples (Fig. 1 d-e, Table S12). Within the mown samples, we detected no overlap between the groups with the exception of four upregulated DEGs in the two field transcriptomes (FbM and FaM (Fig. 1 d). Among these genes were two that could not be annotated, on gene encoding tubulin beta chain 2, and on encoding the GA responsive protein GAST1. Within the non-mown samples, also four genes were shared between the field transcriptomes (FbNM and FaNM)). Among these genes encoding a Chitinase A (class III), expressed only under environmental stress conditions and involved in plant immunity, a Leucin-rich repeat (LRR) family protein, a protein arginine methyltransferase (ATPRMT6), and one gene that we could not annotate. The MADS-box transcription factor-encoding gene *SEP1* was the only gene shared between the field B and the greenhouse (Fig. 1 e). No genes were shared between all three samples, neither in the mown treatment, nor in the non-mown treatment.

#### Genes involved in developmental processes

We were then interested to identify transcriptional changes in genes directing developmental processes required for the regrowth process. Thus, the results of the DEG analysis were restructured such that the DEGs were grouped in 16 descriptive classes defined by database and literature mining (Tables S3 and S10). Those classes (abiotic stress, abiotic/biotic stress, biotic stress, development, general cell function, growth, metabolism, photosynthesis, phytohormone, secondary metabolite biosynthesis, senescence, signaling, symbiosis, transcription, transposon, no available annotation) describe major functional groups and serve to broadly categorize the potential role of a gene (Table S10).

We compared the top 20 DEGs of mown vs. not mown plants and observed that the greenhouse plants displayed more DEGs (classes growth, transcription, and phytohormone) involved in regrowth processes (Fig. 1c, Table S3). Three DEGs involved in growth, two phytohormone genes, and two transcriptional regulators are among the top 20 DEGs, while ten DEGs are related to biotic and abiotic stress in the greenhouse (Table S3). The top 20 DEGs of field A grown plants include four growth related, three development related and five stress-related DEGs. The top 20 DEGs of field B grown plants included only two growth related and six stress-related transcripts. Taken together, the greenhouse grown plants showed most DEGs related to growth, transcription, and phytohormone actions indicative of a regrowth reaction, as they grew under less stressful conditions than the field grown plants, for which stress related DEGs were more dominant (Fig. 1 a-c /Tables 2, 3, 4).

### Phytohormone-related genes

We were interested in the contribution of individual phytohormones to the regrowth reaction in *T. pratense*, as they are known to play a major role in the regulation of development and stress response. We identified DEGs related to phytohormone synthesis, homeostasis, transport, and signaling for all major classes of phytohormones in the datasets. The four phytohormones with the most associated DEGs were: abscisic acid (8 DEGs), gibberellin (8 DEGs), salicylic acid (6 DEGs), and auxin (5 DEGs) (Fig. 1 f). Abscisic acid and salicylic acid are well-known to be involved in response to drought and abiotic/biotic stress, respectively. Auxin is the major phytohormone required for growth and cell division regulation and thus, we expected DEGs related to these phytohormones to be abundant in our analysis. However, unexpectedly, eight DEGs with gibberellin association were found. As gibberellins regulate growth in response to stresses but have so far not been associated with regrowth after biomass loss, we suggest gibberellin as a novel candidate phytohormone to influence the regrowth response.

### Specific transcriptional regulator families are differentially expressed during the regrowth process

**Fig. 2:**
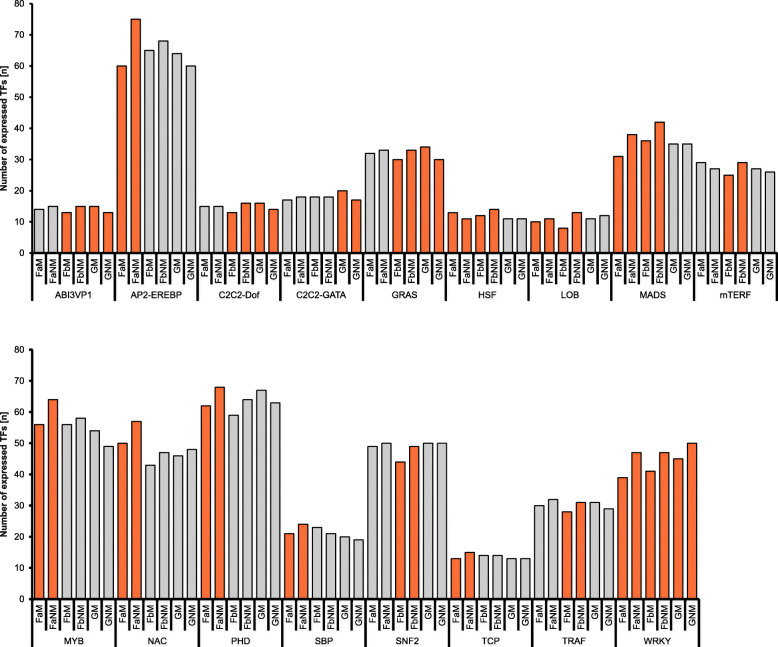
Expressed TRF members in mown and non-mown *T. pratense* plants. The y-axis shows the number of expressed genes (TPM value over 5) that are members of the respective TRF based on PlnTFDB, indicating significant differences in the expression of TRF members upon mowing. Names of the transcriptomes and TRFs are given on the x-axis. Expression of TRF members was compared in a pairwise manner (GM vs GNM, FaM vs FaNM, FbM vs FbNM). Shown are only those plant TRFs in which at least one of the comparisons resulted in a difference of more than 10% of the genes significantly upregulated in either the mown or the unmown condition (orange bars)

As the regulation of stress response, growth and development depends on differential activity of transcription factors, we aimed to identify transcriptional relevant to the biological processes occurring 2 weeks after mowing by mapping the transcriptome to the PlnTFDB [36]. All members of a specific transcriptional regulator family (TRF) were identified in silico and their expression was compared between mown and unmown plants (Table S13). Figure 2 shows TRFs with significantly differential expression between mown and unmown conditions in at least 10% of their members, Fig. S5 and Table S13 includes also those TRFs with 5% of their members regulated differentially upon mowing.

17 TRFs were identified of which at least 10% of the members showed differential expression in mown versus unmown comparisons (Fig. 2): ABI3VP1, AP2-EREBP, C_2_C_2_-Dof, C_2_C_2_-GATA, GRAS, HSF, LOB, MADS, mTERF, MYB, NAC, PHD, SBP, SNF2, TCP, TRAF, WRKY

On field A, the AP2-EREBP, LOB, MADS, MYB, NAC, PHD, SBP, TCP and WRKY TRFs were more prominent in unmown plants, and only the HSF TRFs were more prominent upon mowing. On field B, ABI3VP1, C2C2-Dof, GRAS, HSF, LOB, MADS, mTERF, SNF2, TRAF, and WRKY TRFs were reduced upon mowing. In the greenhouse-grown plants, members of ABI3VP1, C2C2-Dof, C2C2-GATA, and GRAS, show increased numbers in response to mowing. In addition, ARF, C2H2, homeobox, MYB, NAC, and TRAF TRFs show changes in expression in all locations, albeit with only between 5 and 10% of the members being differentially expressed (Table S13).

Two TRFs showed a repression of expression upon mowing: 10% of the WRKY transcripts were less abundant in mown plants regardless of the provenance. In addition, MADS-box transcripts were found upregulated as well, but only in the field-derived transcriptomes. Generally, only four of the 17 TRFs analyzed here showed significant changes in expression towards mowing in the greenhouse-derived plants, suggesting that they react less strongly towards mowing than the field-derived plants. Six TRFs (AP2-EREBP, MYB, NAC, PHD, SBP, and TCP) showed transcriptional changes in reaction to mowing only in field A while only three TRFs (mTERF, SNF2, TRAF) showed this only in field B, suggesting that the combination of biotic and abiotic factors with mowing differed between the two field locations, and, in a similar way, between the field locations and the greenhouse.

Notably, only the C_2_C_2_-GATA TRF showed transcriptional changes in at least 10% of its members towards mowing under greenhouse but not under field-conditions, indicating that transcriptional changes in reaction to other biotic and abiotic factors may overlay the regrowth reaction. Taken together, the TRF analysis showed that the reaction towards mowing induces transcriptional changes in only a subset of TRFs, suggesting that those play a major role in relieving the stress of biomass loss and regrowth.

### Gibberellins are also important regulators after mowing in red clover

We have shown previously (Fig. 1g) that genes related to gibberellins are also differentially expressed, even though GA is not well-known to regulate biological processes related to loss of biomass. We then wanted to know if GA is relevant for the regulation of regrowth and treated red clover plants exogenously with GA_3_.

**Fig. 3:**
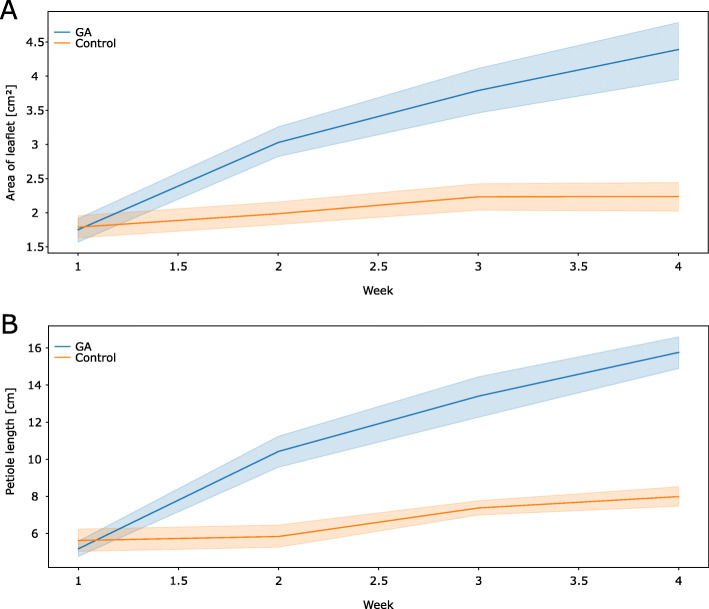
Gibberellin treatment affects regrowth of mown *T. pratense* plants after biomass loss. **a**) The development of leaflet area in cm^2^, **b**) the development of petiole length in cm. Regrowing plants sprayed with GA_3_ showed significantly bigger leaflets and longer petioles. The graphs show average values of the respective plant growth parameters for each sampling date during 4 weeks of observation and the 95% confidence interval. Blue, GA_3_ treated plants; orange, mock-treated plants

A weekly gibberellin application during the regrowth process led to significant and specific changes in morphology (Fig. 3). Previous work suggested that regrowing plants produce smaller and rounder leaflets with shorter petioles than non-mown plants [24]. Thus, number of leaves, shoots and inflorescences, leaf area, and the roundness of leaflets were measured in this experiment (Fig. 3, Fig. S6). The first visible effects of gibberellin treatment were recognized after 1.5 weeks, showing a significant higher leaflet area of gibberellin treated. Later it was observed that the petioles of treated plants were on average twice as long as petioles of untreated plants (16.7 ± 1.9 cm and 8 ± 1.2 cm, respectively). Leaflets of gibberellin treated plants had with 4.7 ± 0.9 cm^2^ almost double the size when compared with those of untreated plants (2.4 ± 0.6 cm^2^). However, gibberellin treated plants produced only 30% more total leaf area than control plants. Other morphological traits such as number of inflorescences, leaves, and shoots remained unaffected by the gibberellin treatment (Fig. S6). In summary, mown plants normally produce leaves with shorter petioles, restrict their leaflet area and their leaves become rounder. Gibberellin treatment partially alleviated these developmental changes such that the mown, gibberellin treated plants produced larger leaves with longer petioles while the leaf shape was unaffected by gibberellin treatment.

## Discussion

### RNA-Seq and assembly

The de novo assembly in combination with a reference-based approach for the annotation led to the identification of 44,643 contigs of which 29,781 were annotated as plant-specific (Fig. S2). With the prior de novo assembly, 4051 additional contigs were obtained that have not been found in the *T. pratense* 1.0 (GCA_000583005.2) genome [12, 35]. The estimated genome size of *T. pratense* is ~ 440 Mbp [27]. The *T. pratense* transcriptome data in this study was ~ 55 Mbp in size, corresponding to ~ 12.5% transcribed regions in the *T. pratense* genome, which is within the range of previously published transcriptomes (~ 10% (42 Mbp) [37]). Interestingly, we found plant-specific, previously unreported contigs suggesting that the *T. pratense* genome might need improvement in terms of sequencing coverage and protein coding sequence annotation.

### Biotic and abiotic stresses contribute to differential gene expression

Plant grown on fields face different stressors when compared to greenhouse grown plants and we were interested in how the field conditions contributed to differential gene expression. The transcriptome comparisons between locations revealed that the mown greenhouse plants showed the highest percentage of DEGs possibly involved in regrowth processes (Fig. 1a-c). In contrast, the field transcriptomes displayed patterns of abiotic and biotic stress reactions. Comparisons of the top 20 DEGs of the unmown field transcriptomes showed that plants grown on field A and B faced more biotic stress than abiotic stress. One of the upregulated genes in field A is a chitinase homolog suggesting that those plants are under attack of fungi and/or insects. Follow-up analyses to correlate environmental conditions, as well as biotic and abiotic stresses monitored within the Biodiversity Exploratories with differential gene expression at the two field locations would be an interesting project but is beyond the scope of this work. In contrast, the top 20 DE transcripts of the greenhouse plants include phytohormone- and transcription-related genes, but also a high proportion of biotic and abiotic stress-related genes. This suggests that also these plants have to cope with stresses, but to a lesser extent emphasizing their regrowth reaction more strongly within the top 20 DEGs. Generally, the non-mown plants show a much higher number of upregulated biotic stress-related genes during a phase in their life when senescence commences and they become more susceptible to pathogen attacks. The mown plants during their regrowth phase are not senescing and their younger organs seem to be less affected by biotic stress.

### Cell walls are remodeled after mowing

After massive biomass loss, like mowing inflicts on *T. pratense*, plants firstly need to seal wounded tissues. Several transcriptional regulators known to play a role in the tissue-reunion processes were identified in *Solanum lycopersicum*, *Cucumis sativus, and A. thaliana* (reviewed in [38]). Homologs of these genes were also identified to be differentially regulated in the *T. pratense* transcriptome after mowing (Table S14), such as several members of the Auxin Response Factor (ARF) family or the No Apical Meristem (NAM) family member *ANAC071,* the homolog of the most highly upregulated transcription factor in greenhouse grown plants after mowing (Table S14) [39]. suggested that high levels of auxin induce the expression of *ANAC071* via ARF6 and ARF8 (in the upper part of incised stems), at the same time, reduced auxin levels directly after the cutting activate the expression of *RAP2.6 L*. In addition auxin signaling via ARF6 and ARF8 influences jasmonic acid synthesis via the activation of *DAD1*, thus together with *LOX2* increases *RAP2.6 L* expression during tissue reunion in *A. thaliana* (Table S14) [39]. Further it was demonstrated that *ANAC071* can initiate the expression of members of the xyloglucan endotransglucosylase/hydrolases family (XTH20 and XTH19) which recombine hemicellulose chains to drive the cell expansion during tissue reunion [40]. Interestingly, we found that all members of the cell wall remodeling pathway show distinct expression patterns- Some are upregulated in mown plants including for example *XTH32* (k69_7012, upregulated in FbM, tdn_94651, upregulated in GM, FaM and FbM), *XTH6* (tdn_91763, upregulated in GM), *XTH8* (k71_5058, upregulated in GM, FbM), *XTH9* (tdn_113578, upregulated in GM), *XTHA* (tdn_87930, upregulated in GM), *LOX2* (tdn_156279, upregulated FbM), and *ARF8* (tdn_156886 upregulated in GM, tdn_156890 upregulated in GM) (Table S9, S13 and S15). This implies that the early steps in the regrowth reaction are conserved in core eudicots and that the cell wall remodeling processes continue at least 2 weeks after mowing.

### Loss of auxin control on axillary buds can be detected two weeks after decapitation

Axillary buds of decapitated *P. sativum* plants export auxin upon growth activation, a process mediated by the *P. sativum* PIN1 homolog PsPIN1. Upon the loss of apical auxin transport, PsPIN1 polarization changes and this new polarization is causing auxin export from dormant axillary buds and is required for their activation [41]. Subcellular targeting and polarization of PsPIN1 starts about 6 h after decapitation and then PsPIN1 expression increases. However, it remains unclear if the elevated PsPIN1 expression is maintained for a prolonged period [42]. Our data show a higher expression of the three PIN1 homologs in greenhouse-grown mown plants when compared to the non-mown control plants (Table S2), indicating a sustained expression of the homolog of PIN1 even after 2 weeks of biomass loss, which might help to activate the remaining dormant buds in *T. pratense.*

### Gibberellin-related genes influence regrowth of *T. pratense* in concert with other phytohormones

Wounding induces a first stress response, activating the interplay of the phytohormones jasmonic acid, salicylic acid, and ethylene. This allows an individual response to various abiotic and biotic stresses, and the differentiation between wounding inflicted by physical forces, pathogens, or herbivory [43–48]. Abscisic acid is required for the fine tuning of the jasmonic acid/salicylic acid/ethylene induced stress response by i.e. suppression of other phytohormones [49]. After the first stress response, additional phytohormones are involved in the regrowth of the plant. Auxin, cytokinin, strigolactone, and gibberellin become involved in a later stage. Following the initiation of shoot outgrowth induced by auxin and cytokinin, an increased gibberellin concentration allows for shoot elongation [31, 50–52]. In addition, auxin, cytokinin, and salicylic acid are involved in the shoot branching, where high levels of auxin and salicylic acid have a suppressing effect on lateral bud outgrowth. High levels of cytokinin promote shoot outgrowth which was shown in *A. thaliana*, *O. sativa*, and *P. sativum* [53–56]. As we were mainly interested in processes that happen approximately 2 weeks after cutting and as the role of those phytohormones was already studied, we concentrated on the role of gibberellin during regrowth, which was also found as one the phytohormones with the most associated genes in our transcriptomes. Gibberellins are involved in multiple aspects of plant development like cell elongation, flowering time regulation, and seed germination. Consequently, genes encoding for proteins involved in the synthesis, perception, and catabolism of the various gibberellins can be assumed to influence plant form. Our RNA-Seq data showed a high abundance of differentially expressed gibberellin associated genes (Fig. 1 F, Table S13)) which may be connected to the morphological changes after mowing, such as rounder leaves, temporary dwarf-like appearance, and higher cumulative biomass production in mown plants [24].

When analyzing the morphological effects of gibberellin application to mown plants (Fig. 3)), external gibberellin application led to the disappearance of specific traits typical for the mowing response. Mown plants developed shorter petioles and their leaf size area was smaller [24], but when treated with gibberellin, leaves and petioles grow up to the size seen in unmown plants.

The cell-expansion and proliferation promoting abilities of gibberellins via stimulation of the degradation of growth-repressing DELLA proteins are well established [57]. The length increase of petioles in gibberellin treated mown plants is in line with reported data from non-mown *Pisum sativum* plants, but in those, leaf sizes remained unchanged after gibberellin treatment [58], suggesting a more specific role for gibberellin in the regrowth reaction after biomass loss in red clover. Moreover, it was shown in *A. thaliana* that elevated gibberellin concentrations enhance cell-division rates in the distal end of leaves (reviewed in [59]). If these results are transferred to *T. pratense*, gibberellin treatment should result in longer leaflets after gibberellin treatment of mown plants. Interestingly, leaf shape did not change, but only the size increased suggesting a regrowth-specific shift of growth pattern which is unaffected by gibberellin but similar to leaf shape of juvenile plants [24]. However, one can also assume a cell-division pattern in red clover leaves that is distinct from the one reported for *A. thaliana* and may react more uniformly to enhance gibberellin concentrations.

Interestingly, gibberellin treatment of mown *T. pratense* plants does not generally lead to stronger longitudinal growth as leaves retained the round shape characteristic for untreated mown plants. These regrowth-specific characteristics can also be found in other species, for example in *A. thaliana*, *Fragaria ananassa*, *Duchesnea indica*, and *G. max*, gibberellin treatment causes elongated petioles and increased leaf sizes and a more erect growth habit [60–63]. This may suggest a new way to increase the accumulation of biomass, suitable for animal fodder. Previous experiments with the grasses *Leymus chinensis* and *Lolium perenne* showed gibberellin action to be limited by N fertilization [64, 65]. Red clover, living in symbiosis with nitrogen fixing bacteria, is not dependent on additional N fertilization and can produce high-protein content biomass without fertilizer on poor soils.

## Methods

### Plant growth conditions, gibberellin treatment, tissue sampling, RNA extraction, cDNA library construction, and RNA-Seq

Plant material for RNA-Seq was collected from three locations (fields and greenhouse, Fig. S1 and Table S1). Field plant tissue for RNA-Seq was sampled on 06.11.2014 within the area of the long-term open research platform Biodiversity Exploratory “Hainich-Dün” [34], located in Thuringia, Germany. Collection permits from farmers and local authorities were obtained centrally by the Biodiversity Exploratory research platform. *T. pratense* is an agriculturally used species native to Germany and does not fall under the Nagoya protocol. ITS sequencing with subsequent database comparisons identified the species collected in the field. The Material was sampled on four neighboring sites; two mown pastures (FaM and FbM) and two meadows that were non-mown (FaNM and FbNM) (Fig. S1 and Table S1). In the year of the sampling, the non-mown pastures and meadows were not grazed upon or mown. The two treatments were comparable as these were the closest experimental plots neighboring one mown and one un-mown plot. The experimental plots were managed as normal agricultural fields and were populated with comparable red clover, as these are wild, established populations. For the greenhouse samples, seeds of regional *T. pratense* populations (from a region covering mainly Thuringia, Saxony, Saxony-Anhalt, Thuringian Forest and Uckermarck, Germany) were obtained from the Rieger Hofmann seed company (Blaufelden, Germany). Plants in the greenhouse were grown in 23 °C with 16 h of light in pots of 12 cm diameter, watered daily, and compound fertilizer (8′8’6′+) was given every 10 days. After 122 days after sowing, half of the plants were cut to 5 cm (GM and GNM). Material from mown plants was sampled approximately 14 days after mowing/cutting, to avoid sequencing of the transcripts related to the first stress response [38]. After collection, the samples were snap frozen in liquid nitrogen.. For each group (FaM, FbM, GM, FaNM, FbNM, and GNM) shoot and leaf material of eight plants was collected. The respective eight plants were separated into two biological replicates of four plants whose RNA was later pooled. As reviewed in [38], the expected time for tissue reunion and wound closure accounts approximately 7 days (cucumber and tomato) to 14 days (*A. thaliana*). Based on this information, we assumed that the first stress response and the initiation of regrowth in *T. pratense* will be approximately 2 weeks after cutting/mowing.

RNA was extracted using NucleoSpin® RNA Plant Kit (Macherey-Nagel GmbH & Co. KG, Düren, Germany) according to the manufacturer’s instructions. For each replicate, equal amounts of RNA of four plants were pooled. Preparation of the cDNA libraries and the strand-specific sequencing were conducted by Eurofins Genomics (Ebersberg, Germany). The RNA-Seq libraries were sequenced on an Illumina Hiseq2000 platform with chemistry v3.0, creating 2 × 100 bp paired end reads.

### Assembly of reference transcriptome and annotation

The raw-read-quality of the RNA-Seq data was analyzed with FastQC (http://www.bioinformatics.babraham.ac.uk/projects/fastqc). Illumina adapter and low quality regions were trimmed using Trimmomatic [42] with ILLUMINACLIP, SLIDINGWINDOW:5:20 and MINLEN:50 options. Quality trimmed reads were pooled and digitally normalized [66]. Multiple de novo assemblies were computed using Trinity [67] and Oases [68] with all odd k-mer parameters between 19 and 85. In addition, a genome guided assembly was performed with Trinity using the draft genome of *T. pratense* 1.0 (GCA_000583005.2) [12, 35]. The resulting contigs were screened for potential coding sequences (CDS) using TransDecoder (https://transdecoder.github.io/). The EvidentialGene pipeline (http://arthropods.eugenes.org/EvidentialGene/about/EvidentialGene _trassembly_pipe.html) was used to merge and filter the contigs based on the TransDecoder CDS prediction. Completeness of the final contig was confirmed by computing the mapping-rate of the non-normalized reads to the contigs. The raw sequence reads can be found at NCBI: PRJNA561285.

The contigs were uploaded to the “Sequence Analysis and Management System” (SAMS) [69] for functional annotation with the SwissProt [70], TrEMBL [71] and Phytozome [72] (e-value cutoff of 1e-5) databases. Additionally, attributes like gene names or functional descriptions were extracted from the blast hits. Contigs were mapped to the *T. pratense* reference genome using gmap [73]. The mapped contigs were compared to the reference annotation of the draft genome. Contigs with similar exon/intron chains (including contained contigs) to a reference gene were assigned the reference gene ID. All non-Viridiplantae contigs were discarded. Transcription factors were identified using a blastp search of the protein sequences against the plant transcription factor database Potsdam (PlnTFDB) ([36], version 3.0 protein database with an e-value cutoff of 1e-20. All functional annotations of transcripts can be found in the Table S2).

### qRT-PCR confirmation of RNA-Seq

qRT-PCR samples included three biological replicates of leaves and axial shoot meristems from common garden experiment-grown plants (for growth conditions of the see Herbert et al. (2018)), as well as the 12 samples used for RNA-Seq. Sample treatment and RNA extraction were performed as described above. First strand cDNA was synthesized by using the RevertAid™ H-Minus First Strand cDNA Synthesis Kit (Thermo Scientific) following the manufacturer protocol.

PerlPrimer software (Marshall, 2004) was used to design qRT-PCR primer (Table S6), and primer efficiency tests were carried out with the same cycler settings, with a standard cDNA dilution series as template. The Roche LightCycler® 480 II system (Roche, Basel, Switzerland) was used for qRT-PCR. The total reaction volume of each sample was 20 μl consisting of 5 μl of 1:50 diluted cDNA template, 1 μl of each primer (10 μM), 3 μl sterile H_2_O, and 10 μl SYBR Green I Master (Roche). The qRT-PCR was carried out with the following cycler settings: 95 °C for 5 min followed by 45 cycles of 95 °C for 10 s, 60 °C for 10 s and 72 °C for 10 s. Contig k65_5754 was used as reference gene [37]. Three biological replicates with two technical replicates were used for each analyzed gene and primer combination. Only primers with an amplification efficiency between 1.8 and 2 were used. For the transcriptome library samples, only two biological replicates were used in the qRT-PCR analysis. Calculations were carried out as described in Pfaffl (2001).

### Differential gene expression analysis, enrichment analysis, and classification of differentially expressed genes

Read counts for each contig of the final assembly in each sample were computed using RSEM [74] with bowtie mapping. To identify differentially expressed (DEGs) *T. pratense* genes, a pairwise comparison of all treatments was preformed using the DESeq2 [75] tool with FDR ≤ 0.01 and |log2foldchange| ≥ 2 between FaM and FaNM, FbM and FbNM; GM and GNM respectively. The top 20 DEGs were determined for each comparison based on the expression strength (log2foldchange). Homologs in the next closest species and *A. thaliana* for each *T. pratense* candidate gene were identified based on the *T. pratense* genome sequence deposited in Phytozome [72]. TPM (transcript per million) values were calculated to estimate contig expression level [76] (for RSEM read counts see Table S16).

We used the description and gene names obtained from TrEMBLE and SwissProt to search the UniProt [77], NCBI [78] and TAIR [79] databases to obtain further information (Table S3). Raw reads that were assembled to contigs, exhibiting a gene structure (ORF) and attained a putative annotation referred to below as genes.

### GO enrichment analysis and Blast2Go analysis of *T. pratense* genomes

To further explore the digital gene expression results and to find more candidate genes/ to identify differentially expressed gene clusters, an enrichment analysis with Gene Ontology (GO) terms [80–82] was performed. For each pairwise comparison, the up-regulated genes were screened for enriched and depleted GO terms using the GOSeq package [83] separately for each treatment. Identified GO terms for each pairwise comparison were then also compared in a mown vs. non-mown manner to show treatment specific GO terms. The results of this analysis were visualized with the program GOplot [84] implemented in RStudio [85] with the program R [86].

Two local BLAST searches [87] with word-size of 3, e-value of 1.0e-3 and HSP length cutoff of 33 were performed against the PlnTFDB using Blast2GO [88]. Only the blast hits with the highest similarity were used for further comparisons (number of BLAST hits = 1), sequences with a similarity below 50% and an e-value higher than 1.0e-4 were omitted. The Blast2GO output was compared with an in-house python3-script utilizing NumPy (https://numpy.org/), Pandas (https://pandas.pydata.org/) and Seaborn (https://seaborn.pydata.org/) applying the list of transcription factors (TF) downloaded from PlnTFDB. We searched the Uniprot database hits for development and phytohormone related genes. Subsequently, we searched for gene IDs of gibberellin genes in our annotated *T. pratense* transcriptomes. Matches were filtered based on TPM values and classified based on biosynthesis and its regulation, catabolism, activation/repression or signaling/response, and the corresponding expression patterns within the transcriptome were identified.

#### Gibberellin treatment

To assess the effect of gibberellin during the regrowth reaction of *T. pratense*, 14 red clover plants were mown as described in [24]. Of these plants, seven were used as control plants and seven plants were sprayed with 100 μM GA_3_ (Duchefa Biochemie B. V, Haarlem, The Netherlands) once per week as described in [89]. Different morphological characters (leaf number, length/width of leaflets, petiole length, number of inflorescences, and number of main shoots) were measured every second day for 4 weeks.

## Supplementary Information

**Additional file 1: Figure S1.** Map of sample locations within the Biodiversity Exploratories.

**Additional file 2: Figure S2.** Annotation Overview: A: Distribution of transcripts that could be mapped to the *T. pratense* genome, to a known locus and were annotated with *T. pratense* genome identifier. B: Distribution of transcripts that could be mapped to an unknown T*. pratense* gene locus. C: Distribution of transcripts that could not be mapped o the *T. pratense* genome. D: Distribution of transcripts of whole transcriptome representing all 12 libraries.

**Additional file 3: Figure S3.** qRT-PCR analysis of selected *T. pratense* genes: A) tdn_146439/ENGase85A (encodes a cytosolic beta-endo-N-acetyglucosaminidase (ENGase), B) tdn_69411/LTP (Lipid transfer protein, a pathogenesis-related protein), C) tdn85889/PME44 (pectin methylesterase 44), D) K65_9861 P5CS (DELTA1-PYRROLINE-5-CARBOXYLATE SYNTHASE 1). Two additional *T. pratense* tissues (axial meristem (AM) and leaves (L)) were included and also the six RNA extractions also used for constructing the transcriptome libraries. Transcriptome data are marked with TPM, all samples are from mown (M) or not mown (NM) plants. Gene expression levels were normalized to the housekeeping contig k65_5754 (not annotated, but with similar expression throughout the transcriptomes). Shown are mean values of the fold change of the respective contig in relation to the expression of contig K65_5754 with error bars that represent standard deviation. On the right side of each graph, the log2 values of the respective gene in each transcriptome are shown for better comparison of expression patterns.

**Additional file 4: Figure S4.** Enrichment analysis of the DEG GO terms. A: Enriched GO terms in mown and non-mown greenhouse samples. B: Enriched GO terms in mown and non-mown field B samples. C: Enriched GO terms in mown and non-mown field A samples. Information about the respective GO number can be found in Table S11.

**Additional file 5: Figure S5.** Expressed transcription factor family members in mown and not mown *T. pratense* plants. The y-axis shows the number of upregulated transcription factors that are members of the respective transcription factor family. Names of the transcriptomes (GM, GNM, FaM, FaNM; FbM, FbNM) and transcription factor families are given on the x-axis. Expression of transcription factor members were compared in a pairwise manner (GM vs GNM, FaM vs FaNM, FbM vs FbNM). Comparisons that resulted in a difference of more than 10% of the contigs significantly upregulated in either the mown or the unmown condition were marked red, differences between 5 and 9 % were marked orange.

**Additional file 6: Figure S6.** Plant architectural characteristics and growth habit of gibberellin treated plants. A-E: Measured, counted or calculated plant characteristics during phenotypic monitoring experiments. Gibberellin treated plants, blue; control plants, orange. Graphs show average values and 95% confidence intervals. Time is shown in weeks. Growth habit of control plants (left side) vs. gibberellin treated plants (right side), after approximately 2 weeks of gibberellin treatment and regrowth (F), and after 4 weeks (G).

**Additional file 7: Table S1.** Overview of the sampling locations for the plant material. Names of the fields belonging to the Biodiversity Exploratory or greenhouse populations are shown. As well as the location, coordinates and conditions (mown/cut and not mown/uncut). **Table S3.** Sources and basis for description and classification of the top 20 DEG. **Table S4.** Number of reads for each sequenced library (transcriptome ID) before and after trimming. **Table S5.** General features of the transcriptome of T. pratense. **Table S6.** Overall alignment rate of the single transcriptomes to the references transcriptome, values above 80% are good. **Table S7.** Annotation of T. pratense plant-specific against different databases. **Table S8.** Quality of the replicates. For each library replicate the number of transcripts above TPM of 1 is shown. Further the number of transcripts shared between to related replica is shown, as well as the number of transcripts unique for a replica. The similarity of the replicas was evaluated by calculating he percentage of the shared transcripts compared to the total number of transcripts of a replica. **Table S10.** Main classes based on the DE contigs. 16 main classes were developed to group the DE contigs. **Table S11.** GO Terms specifically enriched in the individual transcriptomes. **Table S12.** Shared contigs with corresponding annotation. **Table S13.** Detailed information about transcripts described during the discussion part. **Table S14.** Primer sequences.

**Additional file 8: Table S2. **Annotation of the T. pratense transcriptome**.**

**Additional file 9: Table S9. **Analysis of the transcriptomes with Deseq2 of all transcriptome comparisons**.**

**Additional file 10: Table S16. **TPM values for T. pratense transcripts**.**

## Data Availability

The datasets generated and/or analysed during the current study are available in the NCBI database repository, https://www.ncbi.nlm.nih.gov/search/all/?term=PRJNA561285
