# Supplementary figures and images for: Transcriptome analysis reveals major transcriptional changes during regrowth after mowing of red clover (Trifolium pratense)
Source: BMC Plant Biol. 2021 Feb 15;21:95. doi: 10.1186/s12870-021-02867-0 (PMC7885512; doi:10.1186/s12870-021-02867-0)

# Germany

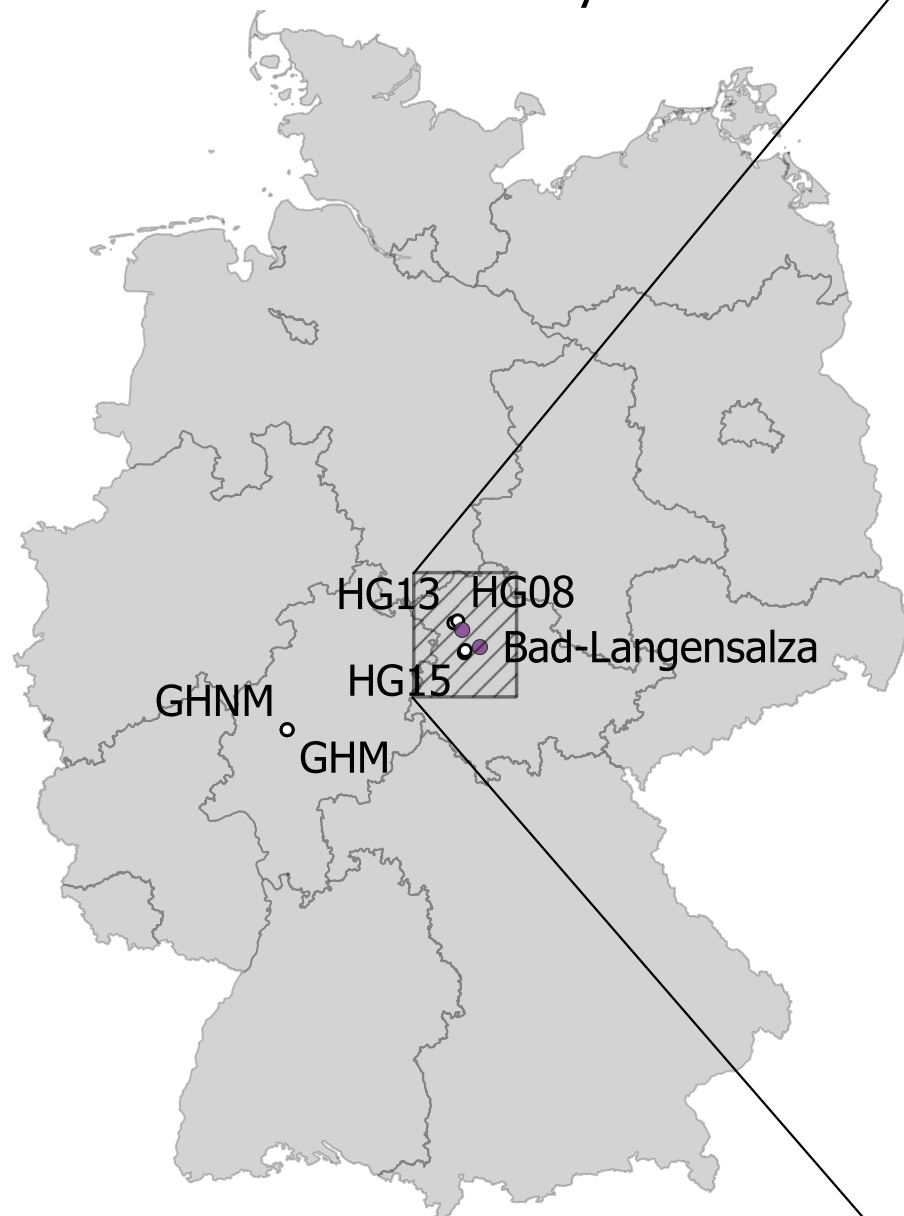

0 180 360 km

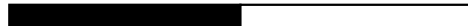

| dist[km] | HG13 | HG08 | HG15 | HG42 |
|----------|------|------|------|------|
| HG13     | 0    | 3.1  | 22.6 | 21.2 |
| HG08     | 3.1  | 0    | 23.1 | 21.8 |
| HG15     | 22.6 | 23.4 | 0    | 1.8  |
| HG42     | 21.2 | 21.8 | 1.8  | 0    |

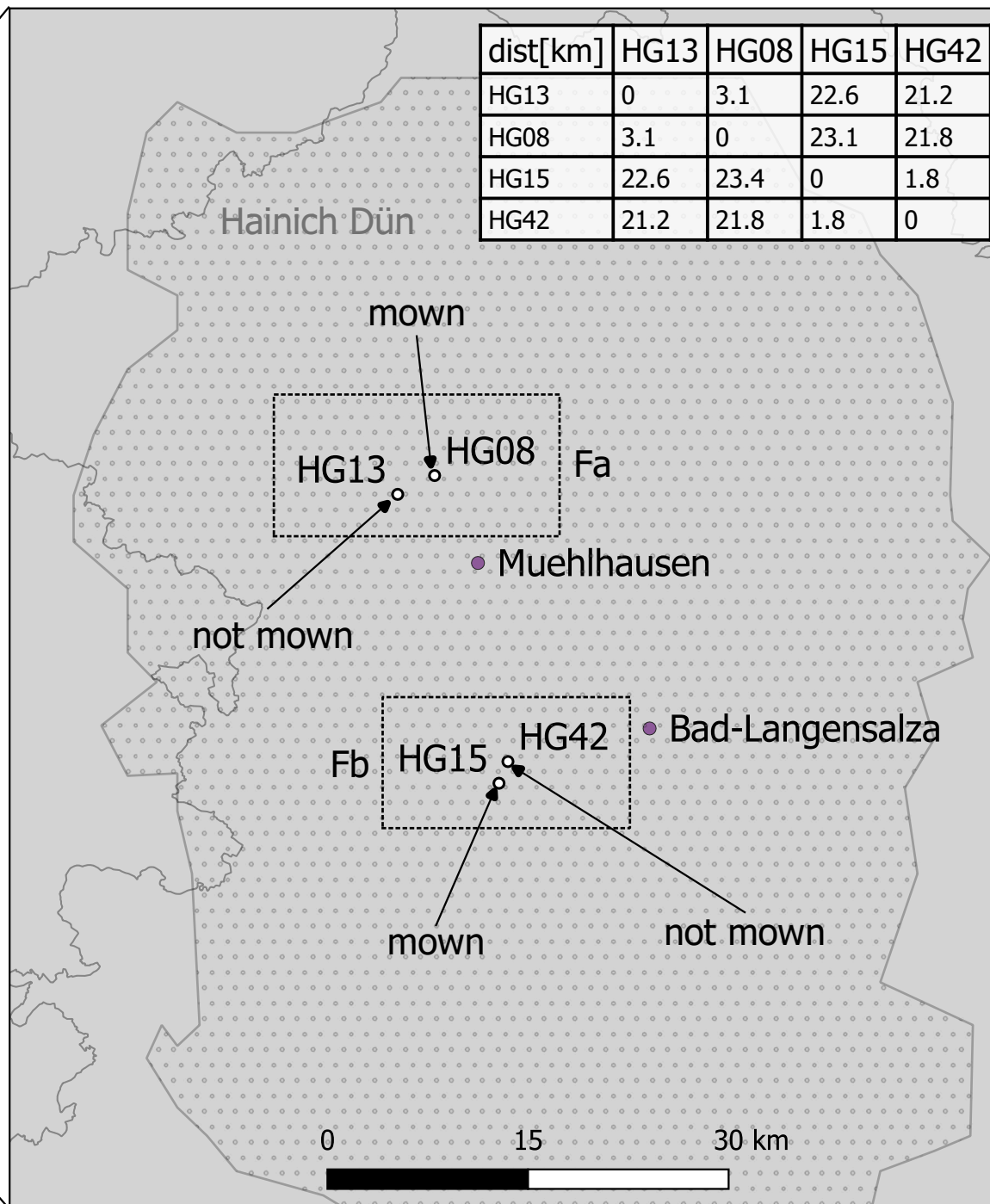

0 15 30 km

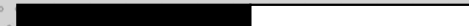

Supplement: Supplementary file 1 — Additional file 1: Figure S1. Map of sample locations within the Biodiversity Exploratories. [file 12870_2021_2867_MOESM1_ESM.pdf]

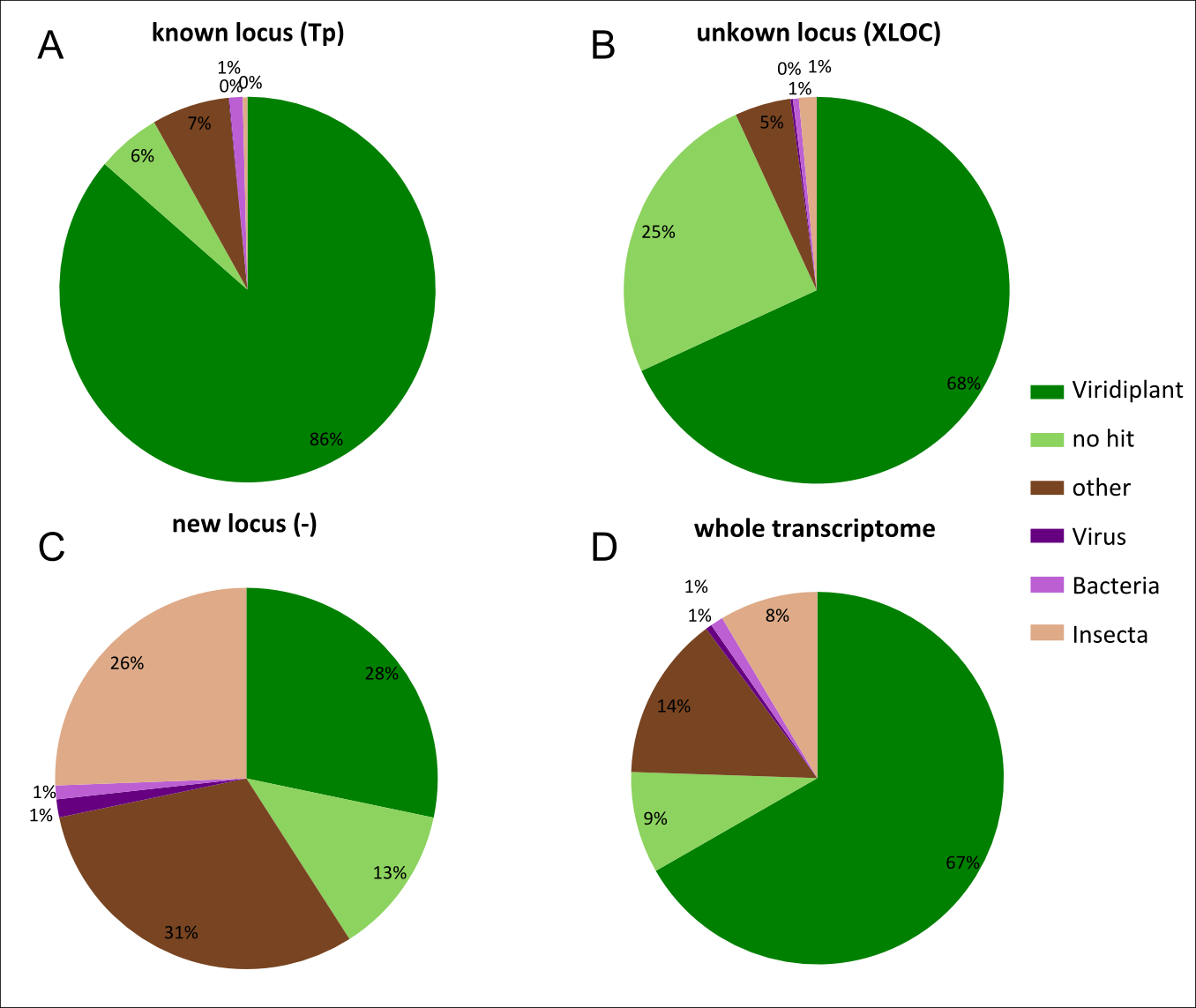

Supplement: Supplementary file 2 — Additional file 2: Figure S2. Annotation Overview: A: Distribution of transcripts that could be mapped to the T. pratense genome, to a known locus and were annotated with T. pratense genome identifier. B: Distribution of transcripts that could be mapped to an unknown T. pratense gene locus. C: Distribution of transcripts that could not be mapped o the T. pratense genome. D: Distribution of transcripts of whole transcriptome representing all 12 libraries. [file 12870_2021_2867_MOESM2_ESM.png]

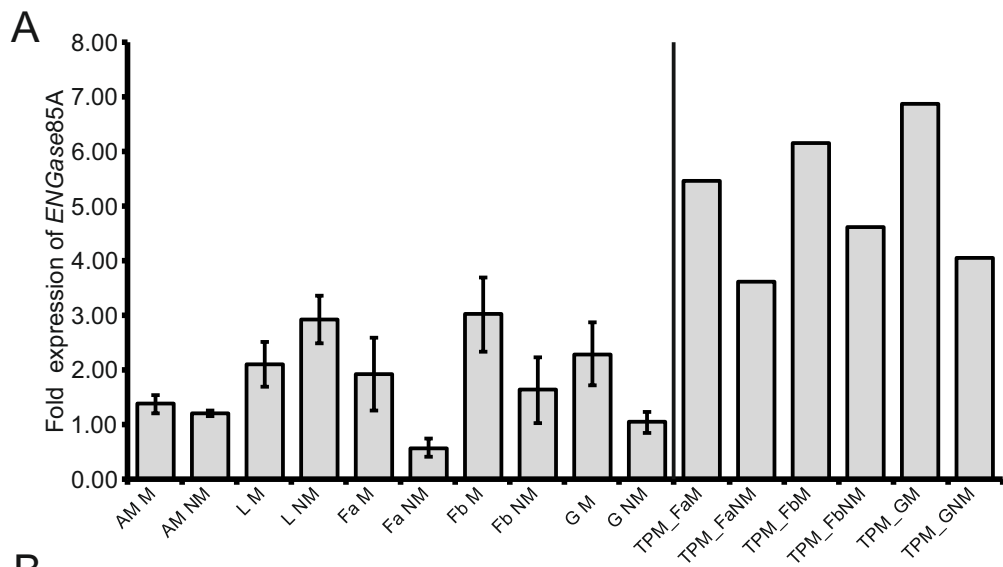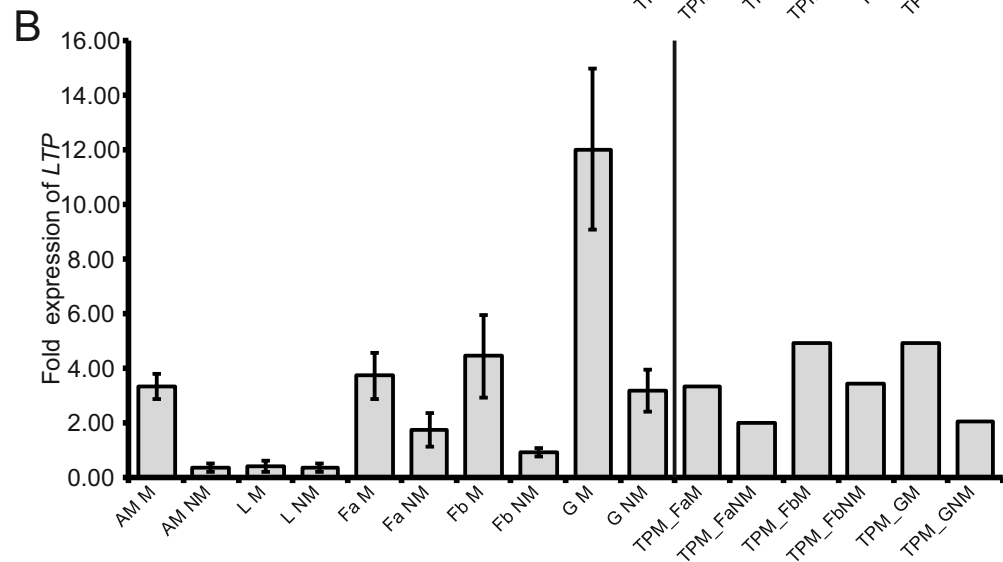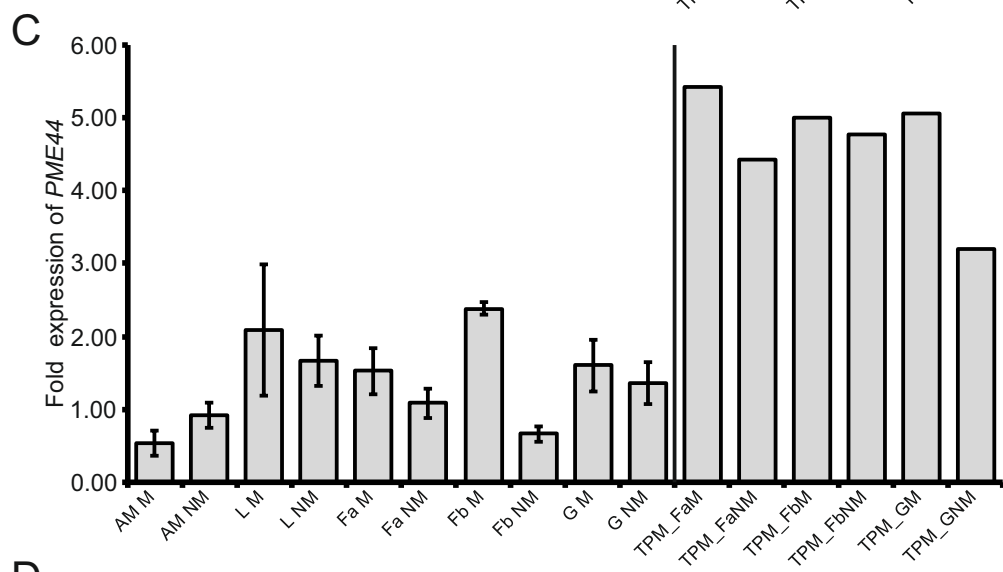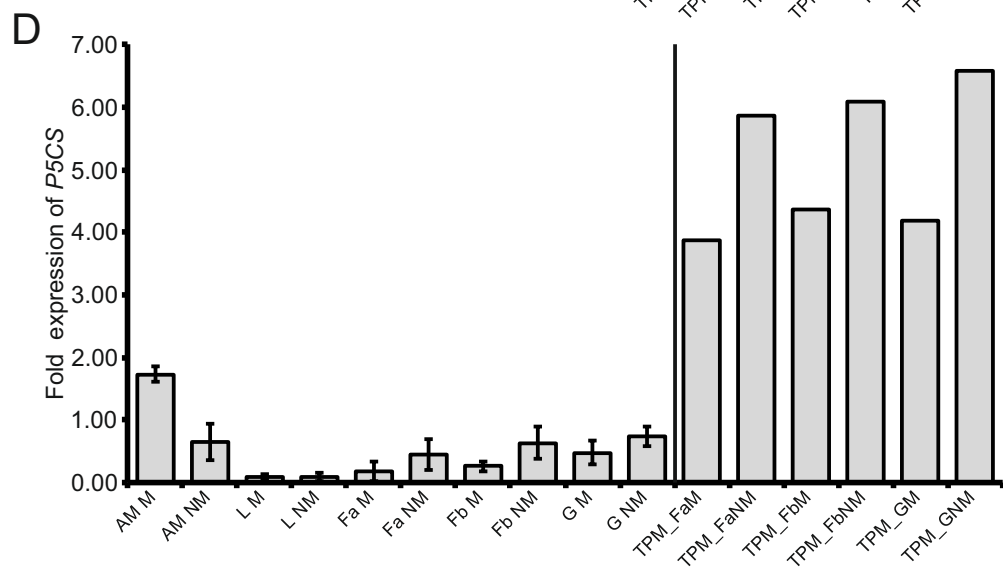

Supplement: Supplementary file 3 — Additional file 3: Figure S3. qRT-PCR analysis of selected T. pratense genes: A) tdn_146439/ENGase85A (encodes a cytosolic beta-endo-N-acetyglucosaminidase (ENGase), B) tdn_69411/LTP (Lipid transfer protein, a pathogenesis-related protein), C) tdn85889/PME44 (pectin methylesterase 44), D) K65_9861 P5CS (DELTA1-PYRROLINE-5-CARBOXYLATE SYNTHASE 1). Two additional T. pratense tissues (axial meristem (AM) and leaves (L)) were included and also the six RNA extractions also used for constructing the transcriptome libraries. Transcriptome data are marked with TPM, all samples are from mown (M) or not mown (NM) plants. Gene expression levels were normalized to the housekeeping contig k65_5754 (not annotated, but with similar expression throughout the transcriptomes). Shown are mean values of the fold change of the respective contig in relation to the expression of contig K65_5754 with error bars that represent standard deviation. On the right side of each graph, the log2 values of the respective gene in each transcriptome are shown for better comparison of expression patterns. [file 12870_2021_2867_MOESM3_ESM.pdf]

A

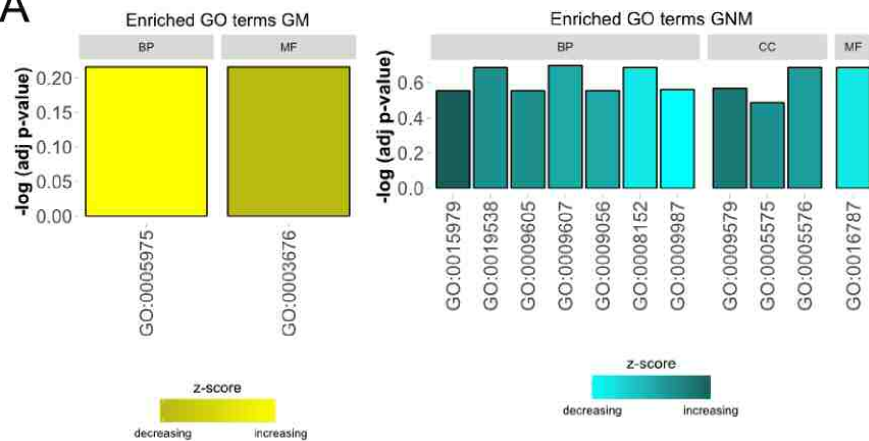

B

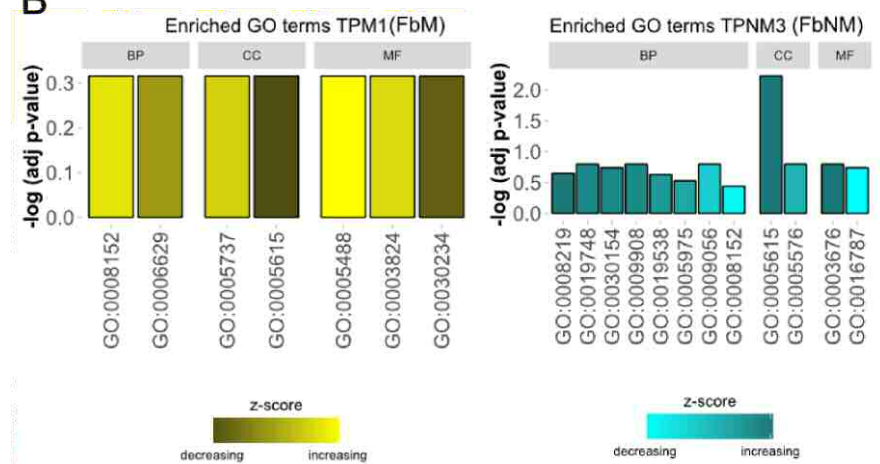

C

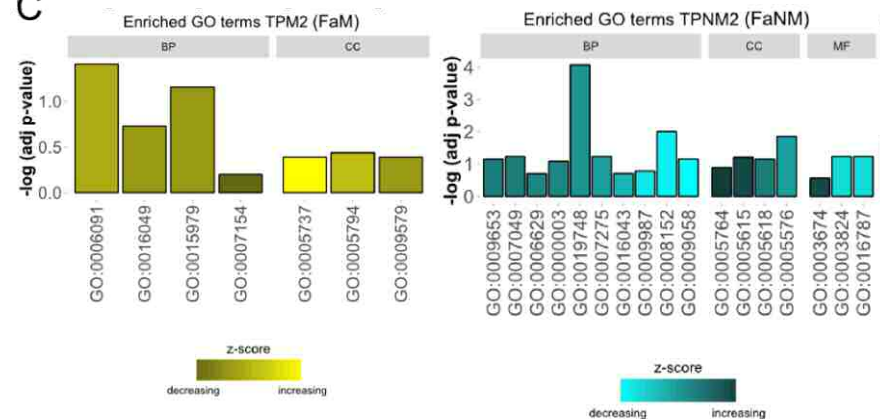

Supplement: Supplementary file 4 — Additional file 4: Figure S4. Enrichment analysis of the DEG GO terms. A: Enriched GO terms in mown and non-mown greenhouse samples. B: Enriched GO terms in mown and non-mown field B samples. C: Enriched GO terms in mown and non-mown field A samples. Information about the respective GO number can be found in Table S11. [file 12870_2021_2867_MOESM4_ESM.pdf]

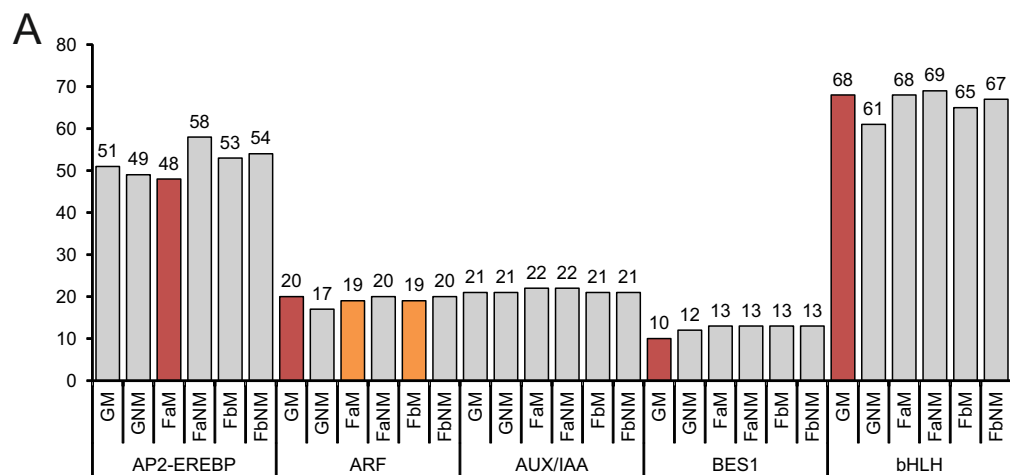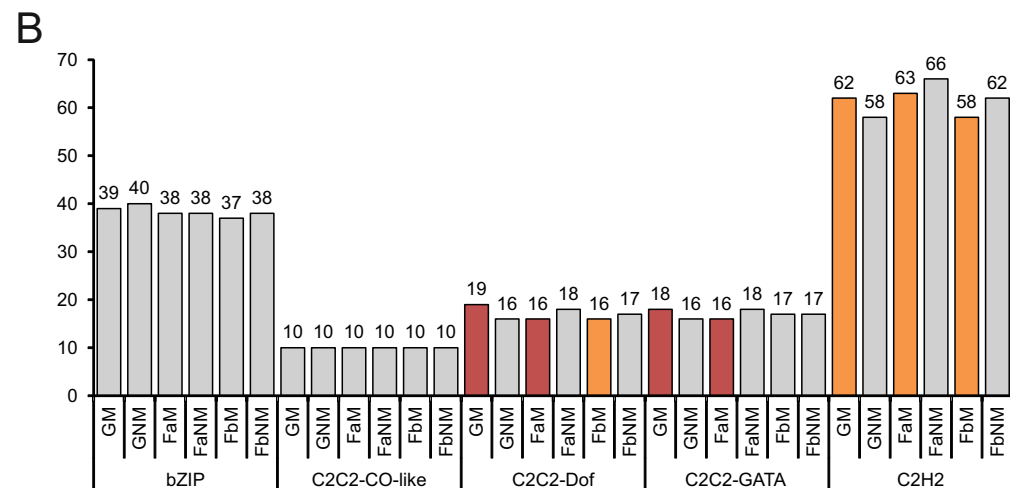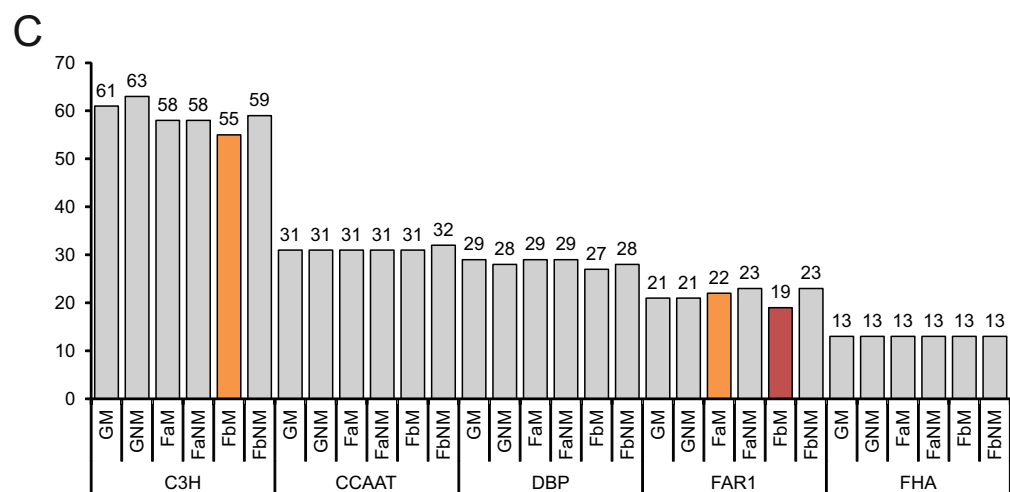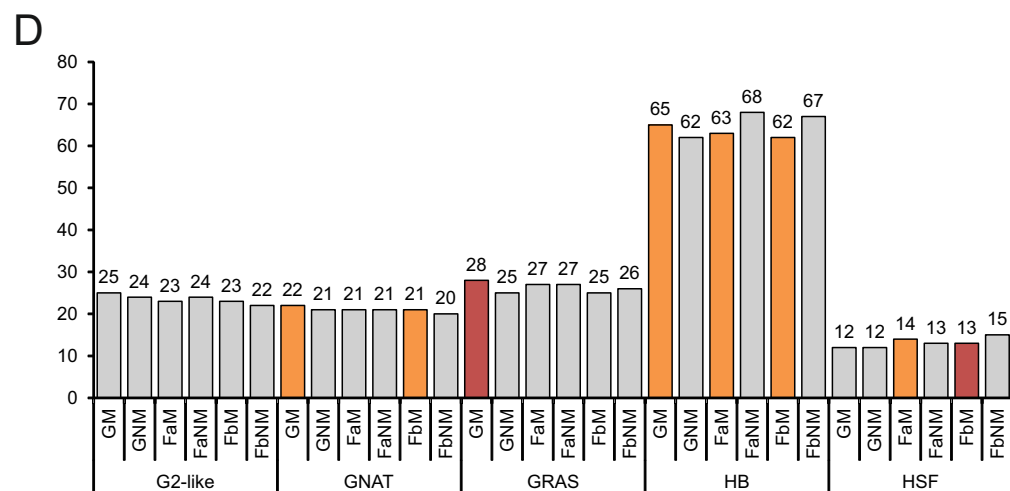

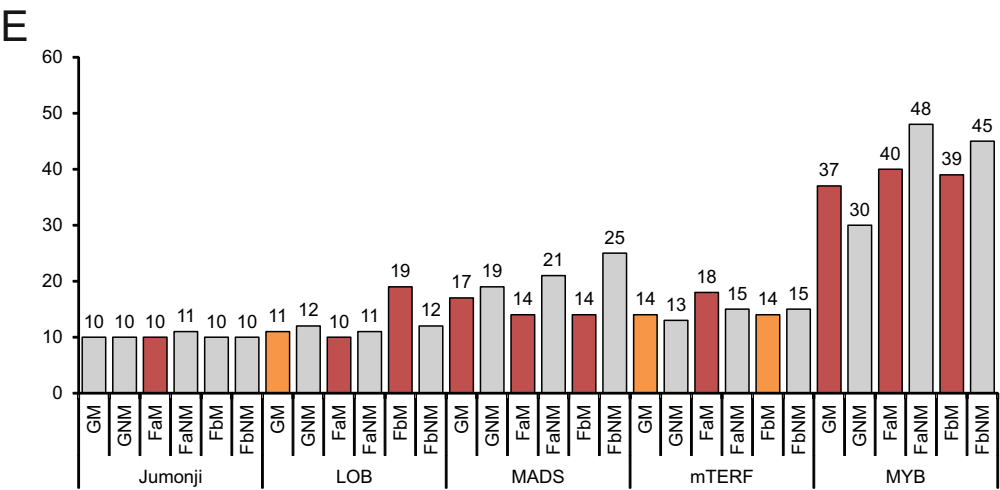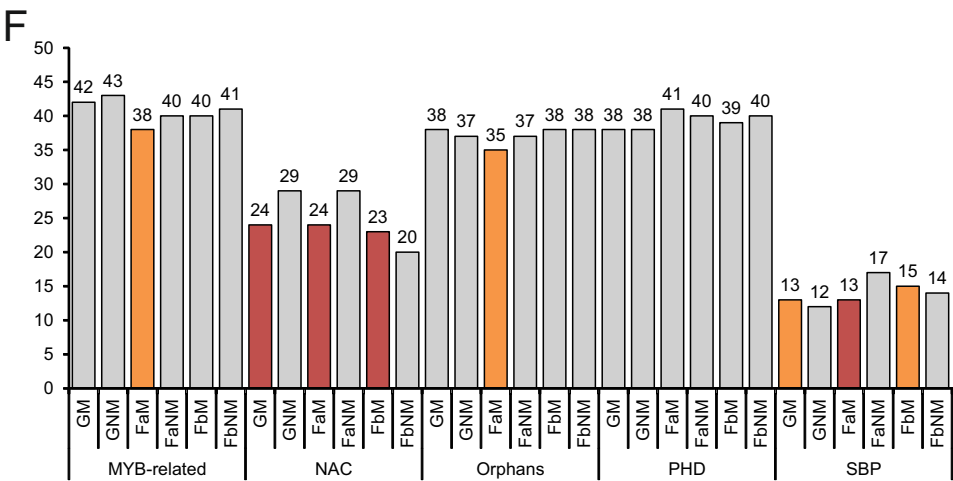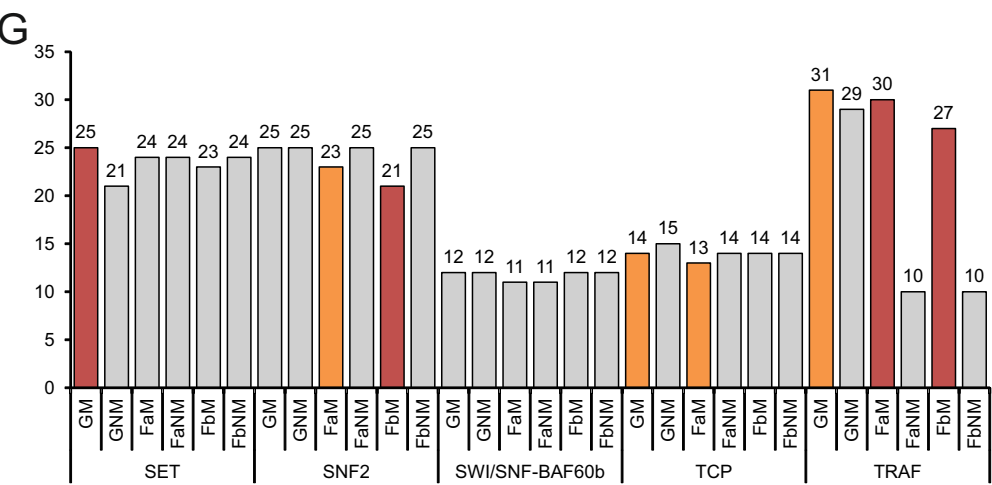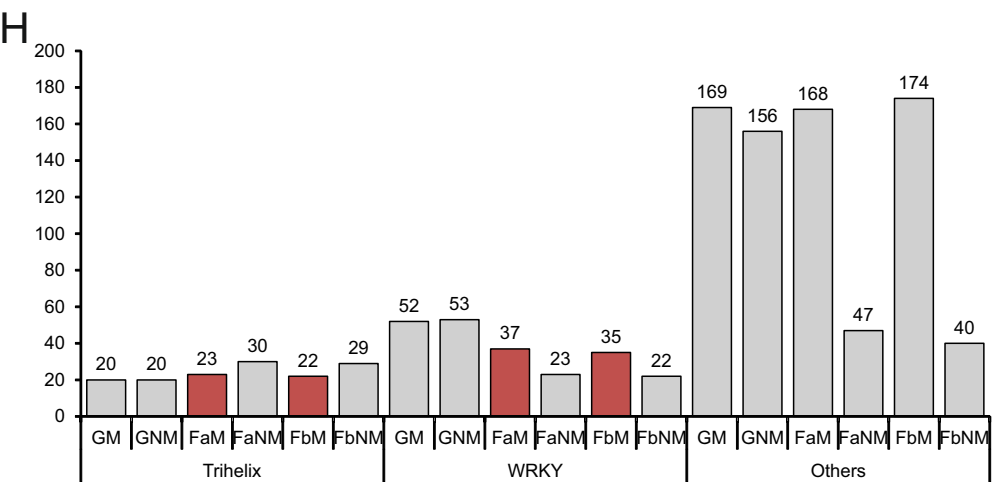

Supplement: Supplementary file 5 — Additional file 5: Figure S5. Expressed transcription factor family members in mown and not mown T. pratense plants. The y-axis shows the number of upregulated transcription factors that are members of the respective transcription factor family. Names of the transcriptomes (GM, GNM, FaM, FaNM; FbM, FbNM) and transcription factor families are given on the x-axis. Expression of transcription factor members were compared in a pairwise manner (GM vs GNM, FaM vs FaNM, FbM vs FbNM). Comparisons that resulted in a difference of more than 10% of the contigs significantly upregulated in either the mown or the unmown condition were marked red, differences between 5 and 9 % were marked orange. [file 12870_2021_2867_MOESM5_ESM.pdf]

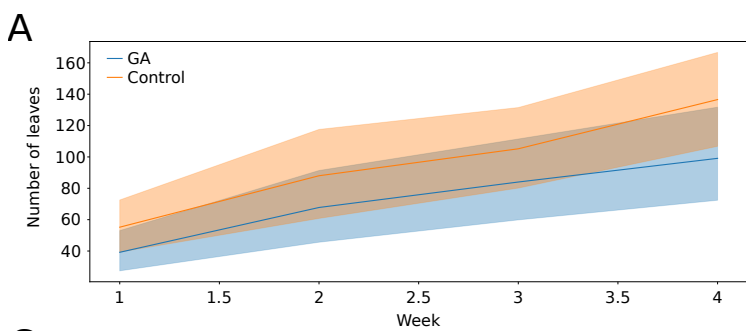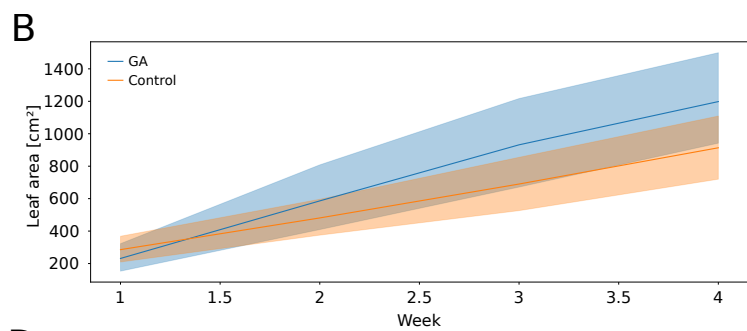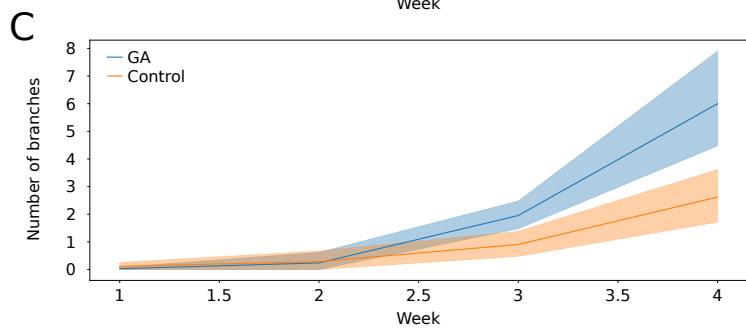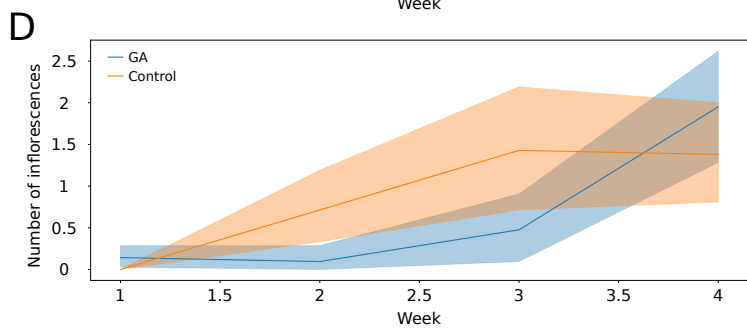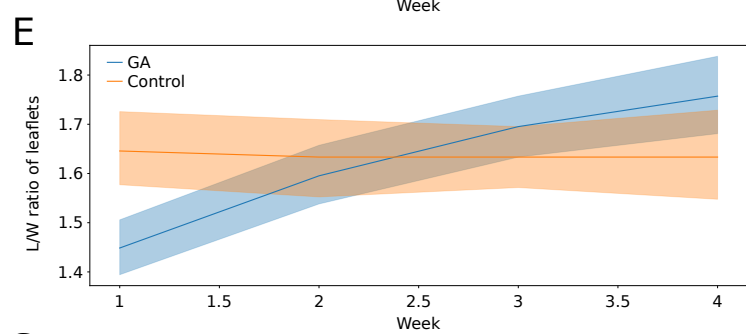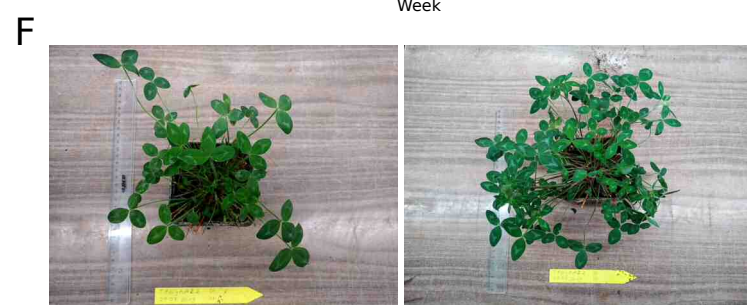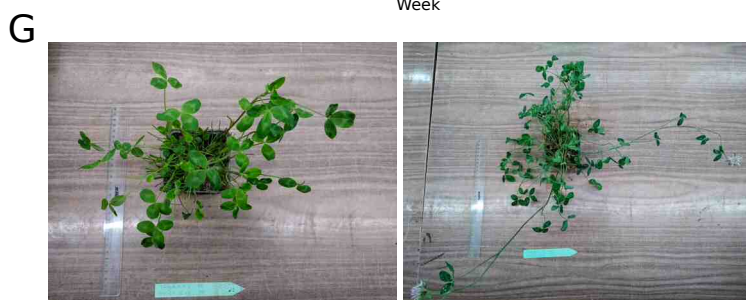

Supplement: Supplementary file 6 — Additional file 6: Figure S6. Plant architectural characteristics and growth habit of gibberellin treated plants. A-E: Measured, counted or calculated plant characteristics during phenotypic monitoring experiments. Gibberellin treated plants, blue; control plants, orange. Graphs show average values and 95% confidence intervals. Time is shown in weeks. Growth habit of control plants (left side) vs. gibberellin treated plants (right side), after approximately 2 weeks of gibberellin treatment and regrowth (F), and after 4 weeks (G). [file 12870_2021_2867_MOESM6_ESM.pdf]
