# Supplementary material for: Transcriptome analysis reveals major transcriptional changes during regrowth after mowing of red clover (Trifolium pratense)
Source: BMC Plant Biol. 2021 Feb 15;21:95. doi: 10.1186/s12870-021-02867-0 (PMC7885512; doi:10.1186/s12870-021-02867-0)
Supplement: Supplementary file 7 — Additional file 7: Table S1. Overview of the sampling locations for the plant material. Names of the fields belonging to the Biodiversity Exploratory or greenhouse populations are shown. As well as the location, coordinates and conditions (mown/cut and not mown/uncut). Table S3. Sources and basis for description and classification of the top 20 DEG. Table S4. Number of reads for each sequenced library (transcriptome ID) before and after trimming. Table S5. General features of the transcriptome of T. pratense. Table S6. Overall alignment rate of the single transcriptomes to the references transcriptome, values above 80% are good. Table S7. Annotation of T. pratense plant-specific against different databases. Table S8. Quality of the replicates. For each library replicate the number of transcripts above TPM of 1 is shown. Further the number of transcripts shared between to related replica is shown, as well as the number of transcripts unique for a replica. The similarity of the replicas was evaluated by calculating he percentage of the shared transcripts compared to the total number of transcripts of a replica. Table S10. Main classes based on the DE contigs. 16 main classes were developed to group the DE contigs. Table S11. GO Terms specifically enriched in the individual transcriptomes. Table S12. Shared contigs with corresponding annotation. Table S13. Detailed information about transcripts described during the discussion part. Table S14. Primer sequences. [file 12870_2021_2867_MOESM7_ESM.docx]

Supplemental tables

Table S1 Overview of the sampling locations for the plant material. Names of the fields belonging to the Biodiversity Exploratory or greenhouse populations are shown. As well as the location, coordinates and conditions (mown/cut and not mown/uncut)

| **Name (replica)** | **ID for RNA-Seq** | **ID for analysis (pooled replicas)** | **Location** | **Estimated coordinates** | **condition** |
| --- | --- | --- | --- | --- | --- |
| **HG13 (HG13a/b)** | TPM2a, TPM2b | Fa(M) | field | N 51°15´35.7"  E 010°22´46.5 | mown |
| **HG08 (HG08a/b)** | TPNM2a, TPNM2b | Fa(NM) | field | N 51°16´20.7"  E 010°25´07.5 | not mown |
| **HG15 (HG15a/b)** | TPM1a, TPM1b | Fb(M) | field | N 51°04´03.7"  E 010°29´13.3 | mown |
| **HG42 (HG42a/b)** | TPNM3a, TPNM3b | Fb(NM) | field | N 51°04´55.8"  E 010°29´47.5 | not mown |
| **GHM (GHMa/b)** | TPGHM1a, TPGHM1b | G(M) | greenhouse | N50°34’10.0’’  E8°40’17.5’’ | cut |
| **GHNM (GHNMa/b)** | TPGHNM1a, TPGHNM1b | G(NM) | greenhouse | N50°34’10.0’’  E8°40’17.5’’ | uncut |

Table S3 Sources and basis for description and classification of the top 20 DEG.

| G top 20 |  |  |  |  |  |
| --- | --- | --- | --- | --- | --- |
| ID | Pattern | Contig ID | log2FoldChange | class | Citation |
| 1 | GHNM | tdn_99733 | -9.5 | growth | [1–4] |
| 2 | GHNM | k41_54584 | -6.3 | biotic stress | [5,6] |
| 3 | GHNM | tdn_92791 | -5.5 | abiotic/biotic stress | [7] |
| 4 | GHNM | k41_130218 | -5.5 | - |  |
| 5 | GHNM | tdn_53091 | -4.8 | phytohormone | [8–12] |
| 6 | GHNM | tgg_43136 | -4.4 | transcription | [13–15] |
| 7 | GHNM | tdn_141837 | -4.3 | abiotic stress | [16–19] |
| 8 | GHNM | tdn_40997 | -4.2 | abiotic stress | [20,21] |
| 9 | GHNM | k71_5292 | -4.1 | biotic stress | [22] |
| 10 | GHNM | k59_6358 | -3.9 | growth | [23–26] |
| 11 | GHM | tdn_86219 | 8.0 | biotic stress | [27,28] |
| 12 | GHM | k23_115785 | 8.0 | abiotic stress | [20,21,29] |
| 13 | GHM | tdn_91159 | 8.1 | biotic stress | [30,31] |
| 14 | GHM | k65_43517 | 8.3 | phytohormone | [32–35] |
| 15 | GHM | tgg_18067 | 8.4 | - |  |
| 16 | GHM | k61_38813 | 9.0 | - |  |
| 17 | GHM | k49_82496 | 9.0 | abiotic/biotic stress | [36] |
| 18 | GHM | k67_38815 | 9.1 | biotic stress | [8,37,38] |
| 19 | GHM | k45_11164 | 9.6 | transcription | [39–41] |
| 20 | GHM | tdn_25484 | 9.6 | growth | [15,42–44] |
|  |  |  |  |  | [1–4] |
| fa top 20 |  |  |  |  |  |
| ID | Pattern | Contig ID | log2FoldChange | Class |  |
| 1 | Fa(NM) | k33_17052 | -9.0 | biotic stress | [45,46] |
| 2 | Fa(NM) | k43_111792 | -8.8 | biotic stress | [47–49] |
| 3 | Fa(NM) | tdn_34568 | -8.6 | - |  |
| 4 | Fa(NM) | tdn_49640 | -8.6 | - |  |
| 5 | Fa(NM) | tdn_58745 | -8.5 | biotic stress | [50,51] |
| 6 | Fa(NM) | tdn_47209 | -8.5 | growth | [52] |
| 7 | Fa(NM) | tdn_48478 | -8.4 | biotic stress | [53,54] |
| 8 | Fa(NM) | k41_17597 | -8.4 | growth |  |
| 9 | Fa(NM) | k51_82581 | -8.2 | growth | [55] |
| 10 | Fa(NM) | tdn_82424 | -8.1 | growth | [55] |
| 11 | Fa(M) | k49_380 | 7.5 | development | [56–58] |
| 12 | Fa(M) | tdn_49869 | 7.6 | - |  |
| 13 | Fa(M) | tdn_54983 | 7.7 | - |  |
| 14 | Fa(M) | k37_9029 | 7.8 | - |  |
| 15 | Fa(M) | k45_6120 | 8.4 | - |  |
| 16 | Fa(M) | k71_23808 | 8.4 | development | [59] |
| 17 | Fa(M) | k59_3541 | 8.4 | development | [60–63] |
| 18 | Fa(M) | k59_360 | 8.6 | metabolism |  |
| 19 | Fa(M) | k53_38903 | 9.0 | abiotic stress | [64–69] |
| 20 | Fa(M) | tdn_129978 | 9.6 | - |  |
|  |  |  |  |  |  |
| Fb top 20 |  |  |  |  |  |
| ID | Pattern | Contig ID | log2FoldChange | Class |  |
| 1 | Fb(NM) | tdn_100726 | -9.4 | biotic stress | [70–72] |
| 2 | Fb(NM) | tgg_49631 | -8.0 | biotic stress | [53,54] |
| 3 | Fb(NM) | tdn_152262 | -7.9 | - |  |
| 4 | Fb(NM) | tdn_56712 | -7.9 | biotic stress | [53,54] |
| 5 | Fb(NM) | tdn_87762 | -7.9 | biotic stress | [73,74] |
| 6 | Fb(NM) | tdn_86129 | -7.1 | general cell functions | [75–77] |
| 7 | Fb(NM) | k55_46241 | -6.9 | Growth | [78] |
| 8 | Fb(NM) | tdn_55533 | -6.2 | abiotic stress | [79–82] |
| 9 | Fb(NM) | tgg_51443 | -4.7 | Growth | [83] |
| 10 | Fb(NM) | tdn_136706 | -4.7 | - |  |
| 11 | Fb(M) | tdn_140636 | 8.8 | general cell functions | [84] |
| 12 | Fb(M) | tdn_154158 | 8.9 | general cell functions | [85–88] |
| 13 | Fb(M) | tdn_65187 | 9.1 | Transposon | [89–93] |
| 14 | Fb(M) | tdn_100956 | 9.2 | Metabolism | [94] |
| 15 | Fb(M) | k63_21505 | 9.3 | biotic stress | [53,54] |
| 16 | Fb(M) | tdn_142681 | 9.3 | secondary metabolite biosynthesis | [95–99] |
| 17 | Fb(M) | k45_6120 | 9.6 | - |  |
| 18 | Fb(M) | tdn_52922 | 10.1 | - | [100–102] |
| 19 | Fb(M) | tdn_65185 | 10.9 | - |  |
| 20 | Fb(M) | tdn_109277 | 11.7 | Transcription | [53,54] |

References for Suppl. Table 3

1. Liu X, Wolfe R, Welch LR, Domozych DS, Popper ZA, Showalter AM. Bioinformatic Identification and Analysis of Extensins in the Plant Kingdom. PLoS ONE. 2016; 11: e0150177. doi: 10.1371/journal.pone.0150177.

2. Bai L, Zhang G, Zhou Y, Zhang Z, Wang W, Du Y, et al. Plasma membrane-associated proline-rich extensin-like receptor kinase 4, a novel regulator of Ca signalling, is required for abscisic acid responses in Arabidopsis thaliana. Plant J. 2009; 60: 314–327. doi: 10.1111/j.1365-313X.2009.03956.x.

3. Lamport DTA, Kieliszewski MJ, Chen Y, Cannon MC. Role of the extensin superfamily in primary cell wall architecture. Plant Physiol. 2011; 156: 11–19. doi: 10.1104/pp.110.169011.

4. Draeger C, Ndinyanka Fabrice T, Gineau E, Mouille G, Kuhn BM, Moller I, et al. Arabidopsis leucine-rich repeat extensin (LRX) proteins modify cell wall composition and influence plant growth. BMC Plant Biol. 2015; 15: 155. doi: 10.1186/s12870-015-0548-8.

5. Zárský V, Kulich I, Fendrych M, Pečenková T. Exocyst complexes multiple functions in plant cells secretory pathways. Curr Opin Plant Biol. 2013; 16: 726–733. doi: 10.1016/j.pbi.2013.10.013.

6. Stegmann M, Anderson RG, Westphal L, Rosahl S, McDowell JM, Trujillo M. The exocyst subunit Exo70B1 is involved in the immune response of Arabidopsis thaliana to different pathogens and cell death. Plant Signal Behav. 2013; 8: e27421. doi: 10.4161/psb.27421.

7. Franz S, Ehlert B, Liese A, Kurth J, Cazalé A-C, Romeis T. Calcium-dependent protein kinase CPK21 functions in abiotic stress response in Arabidopsis thaliana. Mol Plant. 2011; 4: 83–96. doi: 10.1093/mp/ssq064.

8. Sharma M, Pandey GK. Expansion and Function of Repeat Domain Proteins During Stress and Development in Plants. Front Plant Sci. 2016; 6. doi: 10.3389/fpls.2015.01218.

9. Li J, Liu J, Wang G, Cha J-Y, Li G, Chen S, et al. A chaperone function of NO CATALASE ACTIVITY1 is required to maintain catalase activity and for multiple stress responses in Arabidopsis. Plant Cell. 2015; 27: 908–925. doi: 10.1105/tpc.114.135095.

10. Rosado A, Schapire AL, Bressan RA, Harfouche AL, Hasegawa PM, Valpuesta V, et al. The Arabidopsis tetratricopeptide repeat-containing protein TTL1 is required for osmotic stress responses and abscisic acid sensitivity. Plant Physiol. 2006; 142: 1113–1126. doi: 10.1104/pp.106.085191.

11. Greenboim-Wainberg Y, Maymon I, Borochov R, Alvarez J, Olszewski N, Ori N, et al. Cross talk between gibberellin and cytokinin: the Arabidopsis GA response inhibitor SPINDLY plays a positive role in cytokinin signaling. Plant Cell. 2005; 17: 92–102. doi: 10.1105/tpc.104.028472.

12. Lin Z, Ho C-W, Grierson D. AtTRP1 encodes a novel TPR protein that interacts with the ethylene receptor ERS1 and modulates development in Arabidopsis. J Exp Bot. 2009; 60: 3697–3714. doi: 10.1093/jxb/erp209.

13. Zhang J, Huang G-Q, Zou D, Yan J-Q, Li Y, Hu S, et al. The cotton (Gossypium hirsutum) NAC transcription factor (FSN1) as a positive regulator participates in controlling secondary cell wall biosynthesis and modification of fibers. New Phytol. 2018; 217: 625–640. doi: 10.1111/nph.14864.

14. Asahina M, Satoh S. Molecular and physiological mechanisms regulating tissue reunion in incised plant tissues. J Plant Res. 2015; 128: 381–388. doi: 10.1007/s10265-015-0705-z.

15. Pitaksaringkarn W, Matsuoka K, Asahina M, Miura K, Sage-Ono K, Ono M, et al. XTH20 and XTH19 regulated by ANAC071 under auxin flow are involved in cell proliferation in incised Arabidopsis inflorescence stems. Plant J. 2014; 80: 604–614. doi: 10.1111/tpj.12654.

16. Athanasiou K, Dyson BC, Webster RE, Johnson GN. Dynamic acclimation of photosynthesis increases plant fitness in changing environments. Plant Physiol. 2010; 152: 366–373. doi: 10.1104/pp.109.149351.

17. Dyson BC, Allwood JW, Feil R, Xu Y, Miller M, Bowsher CG, et al. Acclimation of metabolism to light in Arabidopsis thaliana: the glucose 6-phosphate/phosphate translocator GPT2 directs metabolic acclimation. Plant Cell Environ. 2015; 38: 1404–1417. doi: 10.1111/pce.12495.

18. Dyson BC, Webster RE, Johnson GN. GPT2: a glucose 6-phosphate/phosphate translocator with a novel role in the regulation of sugar signalling during seedling development. Annals of Botany. 2014; 113: 643–652. doi: 10.1093/aob/mct298.

19. Niewiadomski P, Knappe S, Geimer S, Fischer K, Schulz B, Unte US, et al. The Arabidopsis plastidic glucose 6-phosphate/phosphate translocator GPT1 is essential for pollen maturation and embryo sac development. Plant Cell. 2005; 17: 760–775. doi: 10.1105/tpc.104.029124.

20. Al-Whaibi MH. Plant heat-shock proteins: A mini review. Journal of King Saud University - Science. 2011; 23: 139–150. doi: 10.1016/j.jksus.2010.06.022.

21. Swindell WR, Huebner M, Weber AP. Transcriptional profiling of Arabidopsis heat shock proteins and transcription factors reveals extensive overlap between heat and non-heat stress response pathways. BMC Genomics. 2007; 8: 125. doi: 10.1186/1471-2164-8-125.

22. Lagarda-Diaz I, Guzman-Partida AM, Vazquez-Moreno L. Legume Lectins: Proteins with Diverse Applications. Int J Mol Sci. 2017; 18. doi: 10.3390/ijms18061242.

23. Lee Y, Choi D, Kende H. Expansins: ever-expanding numbers and functions. Curr Opin Plant Biol. 2001; 4: 527–532. doi: 10.1016/S1369-5266(00)00211-9.

24. Cosgrove DJ. Growth of the plant cell wall. Nat Rev Mol Cell Biol. 2005; 6: 850–861. doi: 10.1038/nrm1746.

25. Jiang S-Y, Jasmin PXH, Ting YY, Ramachandran S. Genome-wide identification and molecular characterization of Ole_e_I, Allerg_1 and Allerg_2 domain-containing pollen-allergen-like genes in Oryza sativa. DNA Res. 2005; 12: 167–179. doi: 10.1093/dnares/dsi005.

26. Yennawar NH, Li L-C, Dudzinski DM, Tabuchi A, Cosgrove DJ. Crystal structure and activities of EXPB1 (Zea m 1), a beta-expansin and group-1 pollen allergen from maize. Proc Natl Acad Sci U S A. 2006; 103: 14664–14671. doi: 10.1073/pnas.0605979103.

27. Ketudat Cairns JR, Esen A. β-Glucosidases. Cell Mol Life Sci. 2010; 67: 3389–3405. doi: 10.1007/s00018-010-0399-2.

28. Barrett T, Suresh CG, Tolley SP, Dodson EJ, Hughes MA. The crystal structure of a cyanogenic β-glucosidase from white clover, a family 1 glycosyl hydrolase. Structure. 1995; 3: 951–960. doi: 10.1016/S0969-2126(01)00229-5.

29. Park C-J, Seo Y-S. Heat Shock Proteins: A Review of the Molecular Chaperones for Plant Immunity. Plant Pathol J. 2015; 31: 323–333. doi: 10.5423/PPJ.RW.08.2015.0150.

30. Hothorn M, Wolf S, Aloy P, Greiner S, Scheffzek K. Structural insights into the target specificity of plant invertase and pectin methylesterase inhibitory proteins. Plant Cell. 2004; 16: 3437–3447. doi: 10.1105/tpc.104.025684.

31. Lionetti V, Raiola A, Camardella L, Giovane A, Obel N, Pauly M, et al. Overexpression of Pectin Methylesterase Inhibitors in Arabidopsis Restricts Fungal Infection by Botrytis cinerea1CW. Plant Physiol. 2007; 143: 1871–1880. doi: 10.1104/pp.106.090803.

32. Hoffmann M, Lehmann T, Neu D, Hentrich M, Pollmann S. Expression of AMIDASE1 (AMI1) is suppressed during the first two days after germination. Plant Signal Behav. 2010; 5: 1642–1644. doi: 10.4161/psb.5.12.13810.

33. Sánchez-Parra B, Frerigmann H, Alonso M-MP, Loba VC, Jost R, Hentrich M, et al. Characterization of Four Bifunctional Plant IAM/PAM-Amidohydrolases Capable of Contributing to Auxin Biosynthesis. Plants (Basel). 2014; 3: 324–347. doi: 10.3390/plants3030324.

34. Pollmann S, Neu D, Weiler EW. Molecular cloning and characterization of an amidase from Arabidopsis thaliana capable of converting indole-3-acetamide into the plant growth hormone, indole-3-acetic acid. Phytochemistry. 2003; 62: 293–300.

35. Mano Y, Nemoto K, Suzuki M, Seki H, Fujii I, Muranaka T. The AMI1 gene family: indole-3-acetamide hydrolase functions in auxin biosynthesis in plants. J Exp Bot. 2010; 61: 25–32. doi: 10.1093/jxb/erp292.

36. Kotera E, Tasaka M, Shikanai T. A pentatricopeptide repeat protein is essential for RNA editing in chloroplasts. Nature. 2005; 433: 326–330. doi: 10.1038/nature03229.

37. Sharma N, Sharma KP, Gaur RK, Gupta VK. Role of Chitinase in Plant Defense. Asian J. of Biochemistry. 2011; 6: 29–37. doi: 10.3923/ajb.2011.29.37.

38. Grover A. Plant Chitinases: Genetic Diversity and Physiological Roles. Critical Reviews in Plant Sciences. 2012; 31: 57–73. doi: 10.1080/07352689.2011.616043.

39. Albrecht M, Golatta M, Wüllner U, Lengauer T. Structural and functional analysis of ataxin-2 and ataxin-3. Eur J Biochem. 2004; 271: 3155–3170. doi: 10.1111/j.1432-1033.2004.04245.x.

40. Jiménez-López D, Guzmán P. Insights into the evolution and domain structure of Ataxin-2 proteins across eukaryotes. BMC Res Notes. 2014; 7: 453. doi: 10.1186/1756-0500-7-453.

41. Hecht V, Stiefel V, Delseny M, Gallois P. A new Arabidopsis nucleic-acid-binding protein gene is highly expressed in dividing cells during development. Plant Mol Biol. 1997; 34: 119–124.

42. Hara Y, Yokoyama R, Osakabe K, Toki S, Nishitani K. Function of xyloglucan endotransglucosylase/hydrolases in rice. Annals of Botany. 2014; 114: 1309–1318. doi: 10.1093/aob/mct292.

43. Kaewthai N, Gendre D, Eklöf JM, Ibatullin FM, Ezcurra I, Bhalerao RP, et al. Group III-A XTH genes of Arabidopsis encode predominant xyloglucan endohydrolases that are dispensable for normal growth. Plant Physiol. 2013; 161: 440–454. doi: 10.1104/pp.112.207308.

44. Labavitch JM, Ray PM. Relationship between Promotion of Xyloglucan Metabolism and Induction of Elongation by Indoleacetic Acid 1. Plant Physiol. 1974; 54: 499–502.

45. Richau KH, Kaschani F, Verdoes M, Pansuriya TC, Niessen S, Stüber K, et al. Subclassification and biochemical analysis of plant papain-like cysteine proteases displays subfamily-specific characteristics. Plant Physiol. 2012; 158: 1583–1599. doi: 10.1104/pp.112.194001.

46. Misas-Villamil JC, van der Hoorn RAL, Doehlemann G. Papain-like cysteine proteases as hubs in plant immunity. New Phytol. 2016; 212: 902–907. doi: 10.1111/nph.14117.

47. Gill US, Uppalapati SR, Gallego-Giraldo L, Ishiga Y, Dixon RA, Mysore KS. Metabolic flux towards the (iso)flavonoid pathway in lignin modified alfalfa lines induces resistance against Fusarium oxysporum f. sp. medicaginis. Plant Cell Environ. 2018; 41: 1997–2007. doi: 10.1111/pce.13093.

48. Li X, Chen W, Zhao Y, Xiang Y, Jiang H, Zhu S, et al. Downregulation of caffeoyl-CoA O-methyltransferase (CCoAOMT) by RNA interference leads to reduced lignin production in maize straw. Genet Mol Biol. 2013; 36: 540–546. doi: 10.1590/S1415-47572013005000039.

49. Pakusch A-E, Kneusel RE, Matern U. S-adenosyl-l-methionine:trans-caffeoyl-coenzyme A 3-O-methyltransferase from elicitor-treated parsley cell suspension cultures. Archives of Biochemistry and Biophysics. 1989; 271: 488–494. doi: 10.1016/0003-9861(89)90299-3.

50. Kwon SJ, Jin HC, Lee S, Nam MH, Chung JH, Kwon SI, et al. GDSL lipase-like 1 regulates systemic resistance associated with ethylene signaling in Arabidopsis. Plant J. 2009; 58: 235–245. doi: 10.1111/j.1365-313X.2008.03772.x.

51. Akoh CC, Lee G-C, Liaw Y-C, Huang T-H, Shaw J-F. GDSL family of serine esterases/lipases. Prog Lipid Res. 2004; 43: 534–552. doi: 10.1016/j.plipres.2004.09.002.

52. Kosma DK, Molina I, Ohlrogge JB, Pollard M. Identification of an Arabidopsis fatty alcohol:caffeoyl-Coenzyme A acyltransferase required for the synthesis of alkyl hydroxycinnamates in root waxes. Plant Physiol. 2012; 160: 237–248. doi: 10.1104/pp.112.201822.

53. McHale L, Tan X, Koehl P, Michelmore RW. Plant NBS-LRR proteins: adaptable guards. Genome Biol. 2006; 7: 212. doi: 10.1186/gb-2006-7-4-212.

54. Moffett P, Farnham G, Peart J, Baulcombe DC. Interaction between domains of a plant NBS–LRR protein in disease resistance-related cell death. EMBO J. 2002; 21: 4511–4519. doi: 10.1093/emboj/cdf453.

55. Wu R, Li S, He S, Waßmann F, Yu C, Qin G, et al. CFL1, a WW Domain Protein, Regulates Cuticle Development by Modulating the Function of HDG1, a Class IV Homeodomain Transcription Factor, in Rice and ArabidopsisW. Plant Cell. 2011; 23: 3392–3411. doi: 10.1105/tpc.111.088625.

56. Bundock P, Hooykaas P. An Arabidopsis hAT-like transposase is essential for plant development. Nature. 2005; 436: 282–284. doi: 10.1038/nature03667.

57. Knip M, Pater S de, Hooykaas PJJ. The SLEEPER genes: a transposase-derived angiosperm-specific gene family. BMC Plant Biol. 2012; 12: 192. doi: 10.1186/1471-2229-12-192.

58. Knip M, Hiemstra S, Sietsma A, Castelein M, Pater S de, Hooykaas P. DAYSLEEPER: a nuclear and vesicular-localized protein that is expressed in proliferating tissues. BMC Plant Biol. 2013; 13: 211. doi: 10.1186/1471-2229-13-211.

59. Mantegazza O, Gregis V, Mendes MA, Morandini P, Alves-Ferreira M, Patreze CM, et al. Analysis of the arabidopsis REM gene family predicts functions during flower development. Annals of Botany. 2014; 114: 1507–1515. doi: 10.1093/aob/mcu124.

60. Li H, He Z, Lu G, Lee SC, Alonso J, Ecker JR, et al. A WD40 Domain Cyclophilin Interacts with Histone H3 and Functions in Gene Repression and Organogenesis in ArabidopsisW. Plant Cell. 2007; 19: 2403–2416. doi: 10.1105/tpc.107.053579.

61. Li H, Luan S. The cyclophilin AtCYP71 interacts with CAF-1 and LHP1 and functions in multiple chromatin remodeling processes. Mol Plant. 2011; 4: 748–758. doi: 10.1093/mp/ssr036.

62. Irmisch S, Clavijo McCormick A, Günther J, Schmidt A, Boeckler GA, Gershenzon J, et al. Herbivore-induced poplar cytochrome P450 enzymes of the CYP71 family convert aldoximes to nitriles which repel a generalist caterpillar. Plant J. 2014; 80: 1095–1107. doi: 10.1111/tpj.12711.

63. Nafisi M, Goregaoker S, Botanga CJ, Glawischnig E, Olsen CE, Halkier BA, et al. Arabidopsis Cytochrome P450 Monooxygenase 71A13 Catalyzes the Conversion of Indole-3-Acetaldoxime in Camalexin SynthesisW. Plant Cell. 2007; 19: 2039–2052. doi: 10.1105/tpc.107.051383.

64. Rolletschek H, Weber H, Borisjuk L. Energy Status and Its Control on Embryogenesis of Legumes. Embryo Photosynthesis Contributes to Oxygen Supply and Is Coupled to Biosynthetic Fluxes1. Plant Physiol. 2003; 132: 1196–1206. doi: 10.1104/pp.102.017376.

65. Yu Q-B, Huang C, Yang Z-N. Nuclear-encoded factors associated with the chloroplast transcription machinery of higher plants. Front Plant Sci. 2014; 5. doi: 10.3389/fpls.2014.00316.

66. Ishizaki Y, Tsunoyama Y, Hatano K, Ando K, Kato K, Shinmyo A, et al. A nuclear-encoded sigma factor, Arabidopsis SIG6, recognizes sigma-70 type chloroplast promoters and regulates early chloroplast development in cotyledons. Plant J. 2005; 42: 133–144. doi: 10.1111/j.1365-313X.2005.02362.x.

67. Hajdukiewicz PT, Allison LA, Maliga P. The two RNA polymerases encoded by the nuclear and the plastid compartments transcribe distinct groups of genes in tobacco plastids. EMBO J. 1997; 16: 4041–4048. doi: 10.1093/emboj/16.13.4041.

68. Kremnev D, Strand Å. Plastid encoded RNA polymerase activity and expression of photosynthesis genes required for embryo and seed development in Arabidopsis. Front Plant Sci. 2014; 5. doi: 10.3389/fpls.2014.00385.

69. Kindgren P, Kremnev D, Blanco NE, Dios Barajas López J de, Fernández AP, Tellgren-Roth C, et al. The plastid redox insensitive 2 mutant of Arabidopsis is impaired in PEP activity and high light-dependent plastid redox signalling to the nucleus. Plant J. 2012; 70: 279–291. doi: 10.1111/j.1365-313X.2011.04865.x.

70. El-Awaad I, Bocola M, Beuerle T, Liu B, Beerhues L. Bifunctional CYP81AA proteins catalyse identical hydroxylations but alternative regioselective phenol couplings in plant xanthone biosynthesis. Nat Commun. 2016; 7: 11472. doi: 10.1038/ncomms11472.

71. Liu C-J, Huhman D, Sumner LW, Dixon RA. Regiospecific hydroxylation of isoflavones by cytochrome p450 81E enzymes from Medicago truncatula. Plant J. 2003; 36: 471–484.

72. Wang X. Structure, function, and engineering of enzymes in isoflavonoid biosynthesis. Funct Integr Genomics. 2011; 11: 13–22. doi: 10.1007/s10142-010-0197-9.

73. Fagundes D, Bohn B, Cabreira C, Leipelt F, Dias N, Bodanese-Zanettini MH, et al. Caspases in plants: metacaspase gene family in plant stress responses. Funct Integr Genomics. 2015; 15: 639–649. doi: 10.1007/s10142-015-0459-7.

74. Coll NS, Smidler A, Puigvert M, Popa C, Valls M, Dangl JL. The plant metacaspase AtMC1 in pathogen-triggered programmed cell death and aging: functional linkage with autophagy. Cell Death Differ. 2014; 21: 1399–1408. doi: 10.1038/cdd.2014.50.

75. Matzke M, Kanno T, Daxinger L, Huettel B, Matzke AJM. RNA-mediated chromatin-based silencing in plants. Curr Opin Cell Biol. 2009; 21: 367–376. doi: 10.1016/j.ceb.2009.01.025.

76. Wierzbicki AT, Haag JR, Pikaard CS. Noncoding transcription by RNA polymerase Pol IVb/Pol V mediates transcriptional silencing of overlapping and adjacent genes. Cell. 2008; 135: 635–648. doi: 10.1016/j.cell.2008.09.035.

77. Law JA, Jacobsen SE. Establishing, maintaining and modifying DNA methylation patterns in plants and animals. Nat Rev Genet. 2010; 11: 204–220. doi: 10.1038/nrg2719.

78. Kornberg RD. Chromatin structure: a repeating unit of histones and DNA. Science. 1974; 184: 868–871. doi: 10.1126/science.184.4139.868.

79. Stade K, Ford CS, Guthrie C, Weis K. Exportin 1 (Crm1p) Is an Essential Nuclear Export Factor. Cell. 1997; 90: 1041–1050. doi: 10.1016/S0092-8674(00)80370-0.

80. Haasen D, Köhler C, Neuhaus G, Merkle T. Nuclear export of proteins in plants: AtXPO1 is the export receptor for leucine-rich nuclear export signals in Arabidopsis thaliana. Plant J. 1999; 20: 695–705.

81. Noske A, Weichert W, Niesporek S, Röske A, Buckendahl A-C, Koch I, et al. Expression of the nuclear export protein chromosomal region maintenance/exportin 1/Xpo1 is a prognostic factor in human ovarian cancer. Cancer. 2008; 112: 1733–1743. doi: 10.1002/cncr.23354.

82. Yao Y, Dong Y, Lin F, Zhao H, Shen Z, Chen P, et al. The expression of CRM1 is associated with prognosis in human osteosarcoma. Oncol Rep. 2009; 21: 229–235.

83. Lim EK, Li Y, Parr A, Jackson R, Ashford DA, Bowles DJ. Identification of glucosyltransferase genes involved in sinapate metabolism and lignin synthesis in Arabidopsis. J Biol Chem. 2001; 276: 4344–4349. doi: 10.1074/jbc.M007263200.

84. Tesfaye M, Temple SJ, Allan DL, Vance CP, Samac DA. Overexpression of Malate Dehydrogenase in Transgenic Alfalfa Enhances Organic Acid Synthesis and Confers Tolerance to Aluminum1. Plant Physiol. 2001; 127: 1836–1844.

85. Ardley HC, Robinson PA. E3 ubiquitin ligases. Essays Biochem. 2005; 41: 15–30. doi: 10.1042/bse0410015.

86. Biunno I, Castiglioni B, Rogozin IB, DeBellis G, Malferrari G, Cattaneo M. Cross-species conservation of SEL1L, a human pancreas-specific expressing gene. OMICS. 2002; 6: 187–198. doi: 10.1089/153623102760092788.

87. Biunno I, Cattaneo M, Orlandi R, Canton C, Biagiotti L, Ferrero S, et al. SEL1L a multifaceted protein playing a role in tumor progression. J Cell Physiol. 2006; 208: 23–38. doi: 10.1002/jcp.20574.

88. Zhang N, Yin Y, Liu X, Tong S, Xing J, Zhang Y, et al. The E3 Ligase TaSAP5 Alters Drought Stress Responses by Promoting the Degradation of DRIP Proteins1OPEN. Plant Physiol. 2017; 175: 1878–1892. doi: 10.1104/pp.17.01319.

89. Tzertzinis G, Tabor S, Nichols NM. RNA-dependent DNA polymerases. Curr Protoc Mol Biol. 2008; Chapter 3: Unit3.7. doi: 10.1002/0471142727.mb0307s84.

90. Ahn J-W, Atwell BJ, Roberts TH. Serpin genes AtSRP2 and AtSRP3 are required for normal growth sensitivity to a DNA alkylating agent in Arabidopsis. BMC Plant Biol. 2009; 9: 52. doi: 10.1186/1471-2229-9-52.

91. Kim H-S, Nickoloff JA, Wu Y, Williamson EA, Sidhu GS, Reinert BL, et al. Endonuclease EEPD1 Is a Gatekeeper for Repair of Stressed Replication Forks*. J Biol Chem. 2017; 292: 2795–2804. doi: 10.1074/jbc.M116.758235.

92. Wu Y, Lee S-H, Williamson EA, Reinert BL, Cho JH, Xia F, et al. EEPD1 Rescues Stressed Replication Forks and Maintains Genome Stability by Promoting End Resection and Homologous Recombination Repair. PLoS Genet. 2015; 11: e1005675. doi: 10.1371/journal.pgen.1005675.

93. Majorek KA, Dunin-Horkawicz S, Steczkiewicz K, Muszewska A, Nowotny M, Ginalski K, et al. The RNase H-like superfamily: new members, comparative structural analysis and evolutionary classification. Nucleic Acids Res. 2014; 42: 4160–4179. doi: 10.1093/nar/gkt1414.

94. Rappas M, Niwa H, Zhang X. Mechanisms of ATPases - A Multi-Disciplinary Approach. curr protein pept sci. 2004; 5: 89–105. doi: 10.2174/1389203043486874.

95. Zhao D, Tang W, Hao Z, Tao J. Identification of flavonoids and expression of flavonoid biosynthetic genes in two coloured tree peony flowers. Biochem Biophys Res Commun. 2015; 459: 450–456. doi: 10.1016/j.bbrc.2015.02.126.

96. Wen XC, Han J, Leng XP, Ma RJ, Jiang WB, Fang JG. Cloning and expression of UDP-glucose: flavonoid 3-O-glucosyltransferase gene in peach flowers. Genet Mol Res. 2014; 13: 10067–10075. doi: 10.4238/2014.December.4.1.

97. Sui X, Gao X, Ao M, Wang Q, Yang D, Wang M, et al. cDNA cloning and characterization of UDP-glucose: anthocyanidin 3-O-glucosyltransferase in Freesia hybrida. Plant Cell Rep. 2011; 30: 1209–1218. doi: 10.1007/s00299-011-1029-7.

98. Griesser M, Hoffmann T, Bellido ML, Rosati C, Fink B, Kurtzer R, et al. Redirection of flavonoid biosynthesis through the down-regulation of an anthocyanidin glucosyltransferase in ripening strawberry fruit. Plant Physiol. 2008; 146: 1528–1539. doi: 10.1104/pp.107.114280.

99. Li S, Strid Å. Anthocyanin accumulation and changes in CHS and PR-5 gene expression in Arabidopsis thaliana after removal of the inflorescence stem (decapitation). Plant Physiology and Biochemistry. 2005; 43: 521–525. doi: 10.1016/j.plaphy.2005.05.004.

100. Havecker ER, Gao X, Voytas DF. The diversity of LTR retrotransposons. Genome Biol. 2004; 5: 225. doi: 10.1186/gb-2004-5-6-225.

101. McCue AD, Slotkin RK. Transposable element small RNAs as regulators of gene expression. Trends Genet. 2012; 28: 616–623. doi: 10.1016/j.tig.2012.09.001.

102. Grandbastien MA, Lucas H, Morel JB, Mhiri C, Vernhettes S, Casacuberta JM. The expression of the tobacco Tnt1 retrotransposon is linked to plant defense responses. Genetica. 1997; 100: 241–252.

Table S4 Number of reads for each sequenced library (transcriptome ID) before and after trimming.

| **Transcriptome ID** | **Number of reads before trimming (bp)** | **Number of reads after trimming (bp)** |
| --- | --- | --- |
| **TPGHNM1a** | 44,679,882 | 44,106,908 |
| **TPGHNM1b** | 52,669,329 | 52,139,019 |
| **TPGHM1a** | 57,463,076 | 57,116,250 |
| **TPGHM1b** | 48,727,256 | 48,173,113 |
| **TPNM2a** | 46,901,963 | 39,048,065 |
| **TPNM2b** | 58,548,464 | 50,712,601 |
| **TPNM3a** | 71,179,330 | 62,152057 |
| **TPNM3b** | 58,145,199 | 51,120,501 |
| **TPM1a** | 49,461,360 | 42,936,125 |
| **TPM1b** | 17,393,123 | 14,660,131 |
| **TPM2a** | 48,390,622 | 40,114,769 |
| **TPM2b** | 54,481,408 | 44,674,878 |

Table S5 General features of the transcriptome of *T. pratense*

| **Total number of transcripts** | 44,643 |
| --- | --- |
| **Total number of annotated transcripts** | 41,505 |
| **Min length of transcripts** | 124 (bp) |
| **Max length of transcripts** | 15,551 (bp) |
| **Mean length of transcripts** | 1,171.31 (bp) |
| **Median length of transcripts** | 888 (bp) |
| **N (50)** | 1,656 (bp) |
| **N (90)** | 539 (bp) |

Table S6 Overall alignment rate of the single transcriptomes to the references transcriptome, values above 80% are good.

| **Transcriptome ID** | **Overall alignment rate (%)** |
| --- | --- |
| **TPGHNM1a** | 77.85 |
| **TPGHNM1b** | 79.91 |
| **TPGHM1a** | 81.23 |
| **TPGHM1b** | 82.36 |
| **TPNM2a** | 85.49 |
| **TPNM2b** | 83.64 |
| **TPNM3a** | 87.21 |
| **TPNM3b** | 86.41 |
| **TPM1a** | 85.14 |
| **TPM1b** | 85.66 |
| **TPM2a** | 90.32 |
| **TPM2b** | 87.27 |

Table S7 Annotation of *T. pratense* plant-specific against different databases

| **total plant-specific contigs** | **29,781** |
| --- | --- |
| **PlnTFDB** | 2703 |
| **E.C. number (Swissprot/Tremble)** | 9,781 |
| **GO (Swissprot/Tremble)** | 25,648 |
| **COG** | 28,947 |
| **Gene name(Swissprot/Tremble)** | 16,547 |

**Table S8: Quality of the replicates. For each library replicate the number of transcripts above TPM of 1 is shown. Further the number of transcripts shared between to related replica is shown, as well as the number of transcripts unique for a replica. The similarity of the replicas was evaluated by calculating he percentage of the shared transcripts compared to the total number of transcripts of a replica.**

| Library name | Number of transcripts above TPM 1 | Number of transcripts both replicas have in common | Number of transcripts unique for the replicate | Percentage of common transcripts (Number of common transcripts shared between both replicates/ number of transcripts within one replicate) |
| --- | --- | --- | --- | --- |
| TPGHM1a | 27337 | 25485 | 1852 | 93 |
| TPGHM1b | 27226 |  | 1741 | 94 |
| TPGHNM1a | 26805 | 25093 | 1712 | 94 |
| TPGHNM1b | 27048 |  | 1955 | 93 |
| TPM1a | 27263 | 25033 | 2230 | 92 |
| TPM1b | 27318 |  | 2285 | 92 |
| TPM2a | 27018 | 25224 | 1794 | 93 |
| TPM2b | 27170 |  | 1946 | 93 |
| TPNM2a | 27903 | 25000 | 2903 | 90 |
| TPNM2b | 26825 |  | 1825 | 93 |
| TPNM3a | 28211 | 25603 | 2608 | 91 |
| TPNM3b | 27785 |  | 2182 | 92 |

Table S10 Main classes based on the DE contigs. 16 main classes were developed to group the DE contigs.

| Main class | Definition |
| --- | --- |
| abiotic stress | abiotic stress, includes all genes involved in pathways that are responding to abiotic stress like, salt, temperature (cold and heat), light, mechanical stress, drought stress, water stress. In addition, all genes related to detoxification processes and pathway were also included in this group. Including detoxification, (ROS und detoxification) remove of radicals and other harmful metabolites |
| abiotic/biotic stress | Includes contigs that play a role in both processes |
| biotic stress | biotic stress includes all contigs related to plant defense, pathogen attack, growth in response to pathogen attack, wounding |
| development | development includes contigs related to development of embryos, seeds, flower, reproduction, morphogenesis, organogenesis |
| general cell functions | general cell functions involving processes necessary for normal cell survival, like rRNA processing, ubiquitination (other than signaling), cytoskeleton, transport (other than signaling) |
| growth | growth includes contigs directly involved in growth process, also genes related to cell wall modification processes, secondary cell wall components, cell elongation. |
| metabolism | metabolism unifies all genes related to storage, carbohydrates, energy production, and other catabolic and anabolic processes |
| not available (na) | na includes contigs that have no annotation or a too general annotation which made it impossible to group them in one of those classes |
| photosynthesis | photosynthesis includes contigs related to photosynthesis and chloroplast genes |
| phytohormone | phytohormone includes contigs related to phytohormones pathways, inhibition, synthesis, signal transduction, indirectly or directly phytohormones |
| secondary metabolite biosynthesis | secondary metabolite biosynthesis includes contigs involved in pathways or in the production of metabolites that do not belong to the primary metabolites (carbohydrates, lipids, proteins) |
| senescence | senescence includes contigs related to plant cell death pathways, promoting senescence processes |
| signaling | signaling includes contigs related to processes for signaling like second messengers, cell surface proteins, transmitting or transforming signals |
| symbiosis | symbiosis contigs related to the symbiosis of legume plants with bacteria leading to nodule formation |
| transcription | includes all contigs that encode for transcription factors, other DNA binding proteins and those involved in other aspects of gene expression regulation |
| transposon | transposon includes gene identified as transposons, also domesticated transposons |

Table S11: GO Terms specifically enriched in the individual transcriptomes

| Enriched in | GO ID | Description |
| --- | --- | --- |
| GM | GO:0005975 | carbohydrate metabolic process |
|  | GO:0003676 | nucleic acid binding |
| GNM | GO:0015979 | photosynthesis |
|  | GO:0019538 | protein metabolic process |
|  | GO:0009605 | response to external stimulus |
|  | GO:0009607 | response to biotic stimulus |
|  | GO:0009056 | catabolic process |
|  | GO:0008152 | metabolic process |
|  | GO:0009987 | cellular process |
|  | GO:0009579 | thylakoid |
|  | GO:0005575 | cellular component |
|  | GO:0005576 | extracellular region |
|  | GO:0016787 | hydrolase activity |
| FbM | GO:0008152 | metabolic process |
|  | GO:0006629 | lipid metabolic process |
|  | GO:0005737 | cytoplasm |
|  | GO:0005615 | extracellular space |
|  | GO:0005488 | binding |
|  | GO:0003824 | catalytic activity |
|  | GO:0030234 | enzyme regulator activity |
| FbNM | GO:0008219 | cell death |
|  | GO:0019748 | secondary metabolic process |
|  | GO:0030154 | cell differentiation |
|  | GO:0009908 | flower development |
|  | GO:0019538 | protein metabolic process |
|  | GO:0005975 | carbohydrate metabolic process |
|  | GO:0009056 | catabolic process |
|  | GO:0008152 | metabolic process |
|  | GO:0005615 | extracellular space |
|  | GO:0005576 | extracellular region |
|  | GO:0003676 | nucleic acid binding |
|  | GO:0016787 | hydrolase activity |
| FaM | GO:0006091 | generation of precurser metabolism and energy |
|  | GO:0016049 | cell growth |
|  | GO:0015979 | photosynthesis |
|  | GO:0007154 | cell communication |
|  | GO:0005737 | cytoplasm |
|  | GO:0005794 | Golgi apparatus |
|  | GO:0009579 | thylakoid |
| FaNM | GO:0009653 | anatomical structure morhogen |
|  | GO:0007049 | cell cycle |
|  | GO:0006629 | lipid metabolic process |
|  | GO:0000003 | reproduction |
|  | GO:0019748 | secondary metabolic process |
|  | GO:0007275 | multicellular organism development |
|  | GO:0016043 | cellular component organization |
|  | GO:0009987 | cellular process |
|  | GO:0008152 | metabolic process |
|  | GO:0009058 | biosynthetic process |
|  | GO:0005764 | lysosome |
|  | GO:0005615 | extracellular space |
|  | GO:0005618 | cell wall |
|  | GO:0005576 | extracellular region |
|  | GO:0003674 | molecular function |
|  | GO:0003824 | catalytic activity |
|  | GO:0016787 | hydrolase activity |

Table S12 Shared contigs with corresponding annotation

|  | Transcript ID | Shared | class | Transcript name T. pratense | Phytozome description | Next homolog gene name | Next homolog description | next homolog species | A. thaliana description | A. thaliana Locus | A. thaliana description | A. thaliana other names |
| --- | --- | --- | --- | --- | --- | --- | --- | --- | --- | --- | --- | --- |
| 1 | tdn_175393 | FaM/FbM | growth | XLOC_019311 | - | - | - | - | - | - | - | - |
| 2 | tdn_60472 | FaM/FbM | phyothormone | Tp57577_TGAC_v2_mRNA7542.v2 | giberellin-regulated protein 1-related | Medtr1g025220.1 | GASA/GAST/Snakin, Coexpressed with genes in leaf specific coexpression subnetwork | Medicago truncatula | GAST1 protein homolog 1 | AT1G75750 | GA-responsive GAST1 protein homolog regulated by BR and GA antagonistically. Possibly involved in cell elongation based on expression data The mRNA is cell-to-cell mobile. | GASA1 |
| 3 | tdn_152500 | FaM/FbM | general cell functions | Tp57577_TGAC_v2_mRNA8499.v2 | PTHR11588:SF97 - TUBULIN BETA-4 CHAIN-RELATED tubulin beta-4 chain-related | Medtr4g017630.1 | tubulin beta-1 chain, Coexpressed with genes in roots specific coexpression subnetwork | Medicago truncatula | tubulin beta chain 2 | AT5G62690 | encodes tubulin beta-2/beta-3 chain The mRNA is cell-to-cell mobile. | TUB2 |
| 4 | k45_6120 | FaM/FbM | - | Tp57577_TGAC_v2_mRNA2166.v2 | - | Medtr2g007510.1 | hypothetical protein | Medicago truncatula | - | - | - | - |
| 5 | tdn_110743 | GNM/FbNM | transcription | Tp57577_TGAC_v2_mRNA22030.v2 | MADS box protein | Medtr6g464720.1 | MADS-box transcription factor | Medicago truncatula | K-box region and MADS-box transcription factor family protein | AT5G15800 | Encodes a MADS box transcription factor involved flower and ovule development. Functionally redundant with SEP2 and SEP3 | AGL2, SEP1 |
| 6 | tdn_129843 | FaNM/FbNM | biotic stress | Tp57577_TGAC_v2_mRNA8012.v2 | chintase | Medtr1g099310.1 | chitinase, Coexpressed with genes in roots specific coexpression subnetwork | Medicago truncatula | chitinase A | AT5G24090 | Chitinase A (class III) expressed exclusively under environmental stress conditions. Shown be a plant lysozyme involved in plant immunity. | ATCHIA |
| 7 | tdn_93637 | FaNM/FbNM | development | Tp57577_TGAC_v2_mRNA2969.v2 | leucine-rich repeat (LRR) protein associated with apoptosis in muscle tissue | Medtr4g078535.1 | LRR/extensin | Medicago truncatula | Leucine-rich repeat (LRR) family protein, Highly expressed in root | AT4G28380 | Leucine-rich repeat (LRR) family protein;(source:Araport11) | - |
| 8 | k55_12670 | FaNM/FbNM | transcription | Tp57577_TGAC_v2_mRNA28947.v2 | protein arginine n-methyltransferase 6-related | Phvul.003G001000.1 | K11437 - protein arginine N-methyltransferase 6 [EC:2.1.1.-] (PRMT6) | Phaseolus vulgaris | protein arginine methyltransferase 6 | | protein arginine methyltransferase 6;(source:Araport11) | ATPRMT6 |
| 9 | tdn_152262 | FaNM/FbNM | - | - | - | - | - | - | - | - | - | - |

**Table S13: Detailed information about transcripts described during the discussion part**

|  |  |  |  |  | TPM value average for replicates | | | |  |  |
| --- | --- | --- | --- | --- | --- | --- | --- | --- | --- | --- |
| Gene ID in Text | Contig ID | Uniprot ID | Description | Gene Name | TPGHM | TPGHNM | TPFbM | TPFaM | TPFaNM | TPFbNM |
| ARF6 | tdn_137961 | ARFF_ARATH | Auxin response factor 6 | ARF6 | 31 | 30 | 26 | 27 | 27 | 28 |
|  | k73_3345 | ARFF_ARATH | Auxin response factor 6 | ARF6 | 45 | 48 | 47 | 40 | 51 | 52 |
|  | tdn_129086 | ARFF_ARATH | Auxin response factor 6 | ARF6 | 16 | 13 | 16 | 8 | 17 | 20 |
|  | tdn_137960 | ARFF_ORYSI,ARFF_ORYSJ | Auxin response factor 6 | ARF6 | 38 | 41 | 40 | 30 | 39 | 52 |
|  | tgg_576 | ARFF_ARATH | Auxin response factor 6 | ARF6 | 51 | 51 | 30 | 35 | 39 | 35 |
|  | k61_9598 | ARFF_ARATH | Auxin response factor 6 | ARF6 | 85 | 88 | 50 | 43 | 68 | 64 |
| ARF8 | tdn_156886 | ARFH_ARATH | Auxin response factor 8 | ARF8 | 8 | 4 | 4 | 10 | 12 | 9 |
|  | tdn_156890 | ARFH_ARATH | Auxin response factor 8 | ARF8 | 17 | 9 | 14 | 19 | 23 | 24 |
|  | tdn_156891 | ARFH_ARATH | Auxin response factor 8 | ARF8 | 17 | 11 | 17 | 17 | 20 | 23 |
|  | tdn_156889 | ARFH_ARATH | Auxin response factor 8 | ARF8 | 8 | 4 | 8 | 12 | 11 | 12 |
| *DAD1* | k59_11335 | DAD1_ARATH | Dolichyl-diphosphooligosaccharide--protein glycosyltransferase subunit DAD1 | DAD1 | 8 | 5 | 13 | 14 | 18 | 17 |
| LOX2 | k73_052 | LOX21_SOLTU | Linoleate 13S-lipoxygenase 2-1, chloroplastic | LOX2.1 | 1 | 1 | 14 | 12 | 35 | 13 |
|  | tdn_156279 | LOX21_SOLTU | Linoleate 13S-lipoxygenase 2-1, chloroplastic | LOX2.1 | 129 | 381 | 729 | 479 | 577 | 592 |
|  | tgg_57919 | LOX21_SOLTU | Linoleate 13S-lipoxygenase 2-1, chloroplastic | LOX2.1 | 9 | 6 | 13 | 11 | 19 | 13 |
|  | k69_073 | LOX21_SOLTU | Linoleate 13S-lipoxygenase 2-1, chloroplastic | LOX2.1 | 2 | 1 | 1 | 1 | 7 | 1 |
|  | k39_2212 | sp\|O24370\|LOX21_SOLTU | Linoleate 13S-lipoxygenase 2-1, chloroplastic | LOX2.1 | 0 | 0 | 1 | 1 | 2 | 0 |
|  | tdn_155321 | LOX21_SOLTU | Linoleate 13S-lipoxygenase 2-1, chloroplastic | LOX2.1 | 1 | 1 | 3 | 3 | 7 | 3 |
|  | tgg_67744 | LOX21_SOLTU | Linoleate 13S-lipoxygenase 2-1, chloroplastic | LOX2.1 | 7 | 4 | 10 | 10 | 22 | 11 |
|  | k65_7129 | LOX21_SOLTU | Linoleate 13S-lipoxygenase 2-1, chloroplastic | LOX2.1 | 6 | 5 | 11 | 2 | 1 | 11 |
| XTH20 | tdn_94655 | XTH32_ARATH | Probable xyloglucan endotransglucosylase/hydrolase protein 32 | XTH32 | 16 | 20 | 11 | 32 | 35 | 31 |
| XTH19 | tdn_94653 | XTH32_ARATH | Probable xyloglucan endotransglucosylase/hydrolase protein 32 | XTH32 | 20 | 34 | 14 | 17 | 46 | 17 |
|  | tdn_94649 | XTH32_ARATH | Probable xyloglucan endotransglucosylase/hydrolase protein 32 | XTH32 | 9 | 6 | 22 | 23 | 16 | 13 |
|  | k69_7012 | XTH32_ARATH | Probable xyloglucan endotransglucosylase/hydrolase protein 32 | XTH32 | 7 | 3 | 19 | 11 | 10 | 4 |
|  | tdn_94658 | XTH32_ARATH | Probable xyloglucan endotransglucosylase/hydrolase protein 32 | XTH32 | 13 | 8 | 22 | 22 | 21 | 19 |
|  | tdn_94651 | XTH32_ARATH | Probable xyloglucan endotransglucosylase/hydrolase protein 32 | XTH32 | 17 | 4 | 27 | 18 | 9 | 16 |
|  | tdn_91763 | XTH6_ARATH | Probable xyloglucan endotransglucosylase/hydrolase protein 6 | XTH6 | 135 | 56 | 155 | 175 | 206 | 153 |
|  | tdn_142462 | XTH8_ARATH | Probable xyloglucan endotransglucosylase/hydrolase protein 8 | XTH8 | 6 | 4 | 5 | 7 | 7 | 11 |
|  | k73_13283 | sp\|Q8L9A9\|XTH8_ARATH | Probable xyloglucan endotransglucosylase/hydrolase protein 8 | XTH8 | 28 | 44 | 22 | 11 | 18 | 13 |
|  | k71_5058 | XTH8_ARATH | Probable xyloglucan endotransglucosylase/hydrolase protein 8 | XTH8 | 33 | 13 | 51 | 39 | 40 | 46 |
|  | k73_8912 | XTH9_ARATH | Xyloglucan endotransglucosylase/hydrolase protein 9 | XTH9 | 74 | 49 | 127 | 110 | 177 | 188 |
|  | k71_21706 | XTH9_ARATH | Xyloglucan endotransglucosylase/hydrolase protein 9 | XTH9 | 90 | 49 | 203 | 98 | 119 | 97 |
|  | tdn_113578 | XTH9_ARATH | Xyloglucan endotransglucosylase/hydrolase protein 9 | XTH9 | 48 | 20 | 39 | 46 | 71 | 48 |
|  | tdn_87930 | XTHA_PHAAN | Xyloglucan endotransglucosylase/hydrolase protein A | XTHA | 130 | 50 | 98 | 142 | 196 | 157 |
| EIN3 | tdn_109718 | EIN3_ARATH | Protein ETHYLENE INSENSITIVE 3 | EIN3 | 176 | 194 | 94 | 88 | 128 | 113 |
|  | k47_3584 | EIN3_ARATH | Protein ETHYLENE INSENSITIVE 3 | EIN3 | 114 | 140 | 84 | 68 | 89 | 80 |
| AS1 | tdn_65528 | AS1_ARATH | Transcription factor AS1 | AS1 | 30 | 11 | 57 | 45 | 54 | 68 |
| CYP94C1 | tgg_76356 | C94C1_ARATH | Cytochrome P450 94C1 | CYP94C1 | 3 | 21 | 9 | 7 | 14 | 19 |
|  | tdn_72350 | C94C1_ARATH | Cytochrome P450 94C1 | | CYP94C1 | 0 | 0 | 5 | 4 | 8 |
| ZHD1 | tdn_99073 | ZHD1_ARATH | Zinc-finger homeodomain protein 1 | ZHD1 | 10 | 6 | 18 | 11 | 14 | 22 |
|  | tdn_141786 | ZHD11_ORYSI,ZHD11_ORYSJ | Zinc-finger homeodomain protein 11 | ZHD11 | 20 | 6 | 28 | 20 | 24 | 26 |
| MPT1 | tdn_88349 | MPCP1_ARATH | Mitochondrial phosphate carrier protein 1 | MPT1 | 6 | 5 | 6 | 5 | 5 | 7 |
|  | tdn_74047 | MPCP1_ARATH | Mitochondrial phosphate carrier protein 1 | MPT1 | 3 | 3 | 7 | 6 | 10 | 10 |
| MPT3 | tdn_157438 | MPCP3_ARATH | Mitochondrial phosphate carrier protein 3 | MPT3 | 214 | 165 | 274 | 266 | 320 | 308 |
|  | k65_2241 | MPCP3_ARATH | Mitochondrial phosphate carrier protein 3 | MPT3 | 2 | 3 | 4 | 4 | 9 | 7 |
| MYB44 | k69_17034 | MYB44_ARATH | Transcription factor MYB44 | MYB44 | 62 | 58 | 51 | 35 | 64 | 48 |
|  | tdn_89156 | MYB44_ARATH | Transcription factor MYB44 | MYB44 | 44 | 92 | 49 | 49 | 57 | 41 |
|  | tdn_75969 | MYB44_ARATH | Transcription factor MYB44 | MYB44 | 3 | 2 | 10 | 13 | 9 | 15 |
|  | tdn_112881 | MYB44_ARATH | Transcription factor MYB44 | MYB44 | 3 | 3 | 4 | 2 | 5 | 8 |
|  | k69_14809 | MYB44_ARATH | Transcription factor MYB44 | MYB44 | 10 | 9 | 12 | 7 | 12 | 12 |
| SOC1 | tdn_111649 | SOC1_ARATH | MADS-box protein SOC1 | SOC1 | 8 | 11 | 6 | 15 | 19 | 25 |
|  | tdn_111650 | SOC1_ARATH | MADS-box protein SOC1 | SOC1 | 8 | 12 | 9 | 22 | 38 | 47 |
|  | tdn_111657 | SOC1_ARATH | MADS-box protein SOC1 | SOC1 | 23 | 20 | 32 | 21 | 15 | 28 |
|  | tdn_111659 | SOC1_ARATH | MADS-box protein SOC1 | SOC1 | 17 | 12 | 26 | 25 | 24 | 27 |
| AS2 | tdn_83090 | ASNS2_PEA | Asparagine synthetase | AS2 | 198 | 284 | 39 | 42 | 48 | 45 |

**Table S14: Primer sequences**

| **Transcript** | **Gene** | **Forward Primer** | **Reverse Primer** | **Primer efficiency** |
| --- | --- | --- | --- | --- |
| k65_5754 |  | CCCAAGCAAAGAAGAATTAGGA | TGTATCCGTATCAGCTCCCA | 1.95 |
| tdn_146439 | LTP | CGAACCCAACTTCATCTCCT | CCACTTATCATCTATGTAACCACC | 2.017 |
| k65_9861 | P5CS | CTCCTGATTATATTTGAGTCCCGA | CTGGTATGGCTGAAGTAATGAC | 1.994 |
| tdn_69411 | PME44 | CATCCACCTTCCACAAATTATCC | CCCTTTCCACAATAGTTAAGTACC | 2.006 |
| tdn_85889 | ENGASE1 | AACCGATACAAACTCTCATCAC | GAATCACCAAATCCACTACCA | 1.984 |
